# Unmasking crucial residues in adipose triglyceride lipase for coactivation with comparative gene identification-58

**DOI:** 10.1016/j.jlr.2023.100491

**Published:** 2023-12-20

**Authors:** Natalia Kulminskaya, Carlos Francisco Rodriguez Gamez, Peter Hofer, Ines Kathrin Cerk, Noopur Dubey, Roland Viertlmayr, Theo Sagmeister, Tea Pavkov-Keller, Rudolf Zechner, Monika Oberer

**Affiliations:** 1 Institute of Molecular Biosciences, University of Graz, Graz, Austria; 2 BioTechMed Graz, Graz, Austria; 3 BioHealth Field of Excellence, University of Graz, Graz, Austria

**Keywords:** ATGL, adipose triglyceride lipase, PNPLA2, CGI-58, comparative gene identification-58, ABHD5, coactivation, triacylglycerol hydrolase activity, protein-protein interaction, lipolysis, AlphaFold, protein structure

## Abstract

Lipolysis is an essential metabolic process that releases unesterified fatty acids from neutral lipid stores to maintain energy homeostasis in living organisms. Adipose triglyceride lipase (ATGL) plays a key role in intracellular lipolysis and can be coactivated upon interaction with the protein comparative gene identification-58 (CGI-58). The underlying molecular mechanism of ATGL stimulation by CGI-58 is incompletely understood. Based on analysis of evolutionary conservation, we used site directed mutagenesis to study a C-terminally truncated variant and full-length mouse ATGL providing insights in the protein coactivation on a per-residue level. We identified the region from residues N209-N215 in ATGL as essential for coactivation by CGI-58. ATGL variants with amino acids exchanges in this region were still able to hydrolyze triacylglycerol at the basal level and to interact with CGI-58, yet could not be activated by CGI-58. Our studies also demonstrate that full-length mouse ATGL showed higher tolerance to specific single amino acid exchanges in the N209-N215 region upon CGI-58 coactivation compared to C-terminally truncated ATGL variants. The region is either directly involved in protein-protein interaction or essential for conformational changes required in the coactivation process. Three-dimensional models of the ATGL/CGI-58 complex with the artificial intelligence software AlphaFold demonstrated that a large surface area is involved in the protein-protein interaction. Mapping important amino acids for coactivation of both proteins, ATGL and CGI-58, onto the 3D model of the complex locates these essential amino acids at the predicted ATGL/CGI-58 interface thus strongly corroborating the significance of these residues in CGI-58–mediated coactivation of ATGL.

Intracellular lipolysis, the breakdown of stored triacylglycerol (TG) is essential to maintain energy homeostasis. Adipose triglyceride lipase (ATGL), also termed patatin-like phospholipase 2 (PNPLA2), iPLA2, and desnutrin, plays an important role in lipolysis by hydrolyzing long-chain FA-containing TGs (1, 2, 3). In addition to the hydrolytic function, ATGL also exerts a transacylation function, which generates TG or FA esters of hydroxy FAs by esterification of TG-derived FAs with hydroxy-FAs (3, 4, 5, 6). The hydrolytic activity is stimulated at the protein level by the coactivator protein comparative gene identification-58 (CGI-58), also named abhydrolase domain-containing protein 5 (ABHD5) (5, 7). The stimulatory effect of CGI-58 on ATGL is observed upon direct interaction of both proteins and demonstrates the highest efficiency at approximately equimolar concentrations of enzyme and activator protein (7). ATGL is inhibited by the proteins G0/G1 switch gene 2 (G0S2), hypoxia-inducible lipid droplet-associated protein (HILPDA) and the very recently described microsomal triglyceride transfer protein (8, 9, 10, 11, 12, 13, 14, 15, 16, 17). It is assumed that CGI-58 and G0S2 interact at different binding sites: Lack of ATGL-activity in G0S2-expressing cells could not be rescued with increased CGI-58 concentrations (18). Furthermore, ATGL/G0S2 binding was not affected by CGI-58 in coimmunoprecipitation experiments (18). G0S2 and long-chain acyl-coenzyme A inhibit ATGL’s TG-hydrolytic activity in a noncompetitive manner, and the inhibitory effect of oleoyl CoA on ATGL’s TG hydrolytic activity was not alleviated by CGI-58 (19, 20). Together, these data suggest that different mechanism and interfaces govern the activity and regulation of the enzyme.

The physiological relevance of ATGL was thoroughly studied in different genetic KO and transgenic mouse models: mice lacking ATGL exhibit increased TG deposition in many tissues and disrupted signaling pathways leading to disturbed energy homeostasis (21, 22). A comprehensive record of published ATGL mouse models and associated phenotypes was rigorously summarized elsewhere (23). Human patients with mutations in the gene coding for ATGL suffer from TG accumulation in leukocytes and multiple tissues in addition to severe cardiomyopathy (22). Interestingly, mutations in the human gene of the ATGL coactivator CGI-58 cause severe hepatic steatosis and systemic TG accumulation that is always associated with ichthyosis (7, 24). Analogously, global CGI-58 KO mice suffer from a lethal skin permeability barrier defect due to impaired ω-O-acylceramide synthesis (21, 25, 26, 27).

Atomic resolution structures of both proteins, CGI-58 and ATGL, are not available yet. Mouse ATGL (mATGL, 486 amino acids) harbors a “PNPLA domain” within resides I10-K179 (InterPro IPR002641), which is name-giving for all PNPLA-family members (28, 29). The TG-hydrolytic activity of ATGL is catalyzed by a catalytic dyad, formed by S47 and D166, and residues G14-G19 forming the oxyanion hole (30). Residues I10-G24 are suggested to be involved in TG binding (31). In vitro studies showed increased lipolytic activity of the truncated variants of ATGL (truncated after D288 or L254 in mATGL) compared to the WT enzyme (5, 10). Nevertheless, the exact roles of the C-terminal half of ATGL (residues P260-C486) with respect to lipid droplet (LD) localization and autoregulatory function remain to be established.

Mouse CGI-58 (mCGI-58, 351 amino acids) is a member of the α/β-hydrolase-fold containing protein family comprising of an N-terminal region and an α/β-hydrolase core domain with a cap (32). In contrast to other α/β-hydrolase protein family members, CGI-58 does not exhibit hydrolytic activity due to the lack of a catalytic nucleophile. Previously, we have shown that the N-terminal, Trp-rich region of CGI-58 is important for LD anchoring and largely disordered (33, 34, 35). Removal of 30 N-terminal amino acids of mCGI-58 disrupted its ability to localize to LDs and its ability to coactivate ATGL. However, this LD anchor by itself lacks the ability to activate ATGL, indicating that other regions of CGI-58 are necessary for ATGL coactivation (34). Subsequent mutagenesis studies of CGI-58 (ABHD5) demonstrated a crucial function for residues R299, G328, and D334 of CGI-58 in ATGL coactivation (16, 36).

In the current study, we identified evolutionary less conserved parts of ATGL by comparing mammalian ATGL with different phyla in the Kingdom Animalia. Importantly, ATGL variants at positions N209, I212, and N215 exhibited intact or marginally reduced basal activity, however these variants exhibited drastically reduced activatability by CGI-58 providing evidence that these residues play a significant role in the coactivation process. Artificial intelligence–based modeling approaches for the 3D structure of the ATGL/CGI-58 complex also predicted the region comprising N209, I212, and N215 to be involved in protein-protein interaction between enzyme and coactivator. Residues R299, G328, and D334 of CGI-58, are also located in the predicted ATGL/CGI-58 binding interface. In the absence of experimental complex structures, our data provide a good working model of the ATGL/CGI-58 complex.

## Materials and methods

### Materials

If not stated otherwise, chemicals were obtained from Merck (Darmstadt, Germany) or Carl Roth GmbH (Karlsruhe, Germany); columns for protein purification were obtained from Cytiva (formerly GE Healthcare Life Sciences (Uppsala, Sweden)). [9,10-^3^H(N)]-triolein was obtained from PerkinElmer Life Sciences (Waltham, MA, USA). Pierce™ Unstained Protein MW Marker from Thermo Fisher Scientific™ was used as size marker for SDS-PAGE gels. Blue Prestained protein standard broad range marker from New England BioLabs was used for SDS-PAGE and for immuno blotting. Disruption of cells was carried out using a homogenizer (SONOPLUS ultrasonic homogenizer HD 2070, Bandelin, Berlin, Germany).

### Site-directed mutagenesis of bacterial expression vectors

The WT variants coding for *Mus musculus* ATGL (UniProt Accession: Q8BJ56) in two different expression vectors namely pST44 and pcDNA4/HisMaxC (Thermo Fisher Scientific, Waltham, USA) were described in (37). The primers listed in Tables 1 and 2 were designed to introduce point mutations in the WT variants using the Q5® site-directed mutagenesis kit (New England BioLabs, Ipswich, USA). All variants were verified by Sanger sequencing (Microsynth, Balgach, Switzerland).

**Table 1:** Primers for point mutations in mATGL288 and for mCGI-58/mATGL288

Variant	Forward Primer 5′-3′	Reverse Primer 5′-3′	Vector
mCGI58/mATGL288_ N209A	GCGTGTGACGGCTACCAGTATCC	AGCTCGTGGATATTGGTC	pST44
mCGI58/mATGL288_ I212A	GAATACCAGTgccCAGTTCAATCTGCGC	GTCACACGCAGCTCGTGG	pST44
mCGI58/mATGL288_I212S	GAATACCAGTagcCAGTTCAATCTGC	GAATACCAGTagcCAGTTCAATCTGC	pST44
mCGI58/mATGL288_N215A	TATCCAGTTCgctCTGCGCAACTTGTAC	CTGGTATTCGTCACACGC	pST44
S47A of mATGL288-Str	TTATGGTGCGGCGGCAGGCGCCC	ATGTGCGTAGCGTTGGCCAC	pST44
mATGL288_ N209A	TCGCGTCACCgccACCAGCATCC	AGCTCGTGGATGTTGGTG	pST44
mATGL288_ I212A	CAACACCAGCgccCAGTTCAACCTTCG	GTGACGCGAAGCTCGTGG	pST44
mATGL288_ I212S	CAACACCAGCagcCAGTTCAACC	GTGACGCGAAGCTCGTGG	pST44
mATGL288_ N215A	CATCCAGTTCgccCTTCGCAATCTCTACC	CTGGTGTTGGTGACGCGA	pST44
mATGL288_N209A/I212A/N215A	AGTGCTCAGTTCGCTGCGCAAC	GGTAGCCGTCACACGCAGCTCGTG	pST44
mATGL288_L81S	CAAACGGTTTTCGGGACCGCTGC	CGCGCTTCTTTCGATACTTC	pST44
mATGL288_L84A	TCTGGGACCGGCGCATCCGTCCTTTAAC	ACCCGTTTGCGCGCTTCT	pST44
mATGL288_Y164A	AGGCGTACGTGCCGTAGATGGAG	TGCAGAGTCGGCGGAATC	pST44
mATGL288_L205A	TATCCACGAGGCGCGTGTGACGAATAC	TTGGTCGATGAGTCCTGC	pST44
mATGL288_L216A	CCAGTTCAATGCGCGCAACTTGTAC	ATACTGGTATTCGTCACAC	pST44
mATGL288_R221A	CAACTTGTACGCCTTATCCAAAGCCC	CGCAGATTGAACTGGATAC	pST44
mATGL288_L226S	ATCCAAAGCCTCGTTTCCGCCGG	AAGCGGTACAAGTTGCGC	pST44
mATGL288_S188A	TAGTCCCTTTGCCGGTGAAAGCG	ACCGTAATGGTGTTCTTC	pST44
mATGL288_I193A	TGAAAGCGACGCCTGTCCGCAGG	CCGCTAAAGGGACTAACC	pST44
mATGL288_F227A	CAAAGCCCTGGCTCCGCCGGAAC	GATAAGCGGTACAAGTTGC	pST44
mATGL288_F187A	GGTTAGTCCCGCGAGCGGTGAAAG	GTAATGGTGTTCTTCAGC	pST44
mATGL288_Y151A	CTTCATTCCCGTGGCGTGTGGCTTGATTC	GTGGAACAGACGTTC	pST44
mATGL288_Y220A	CGCAACTTGGCGCGCTTATCCAAAG	GATTGAACTGGATACTG	pST44
mATGL288_Y242A	CAAACAGGGTGCGCGTGACGGGTTAC	CACATTTCGCGTAACACC	pST44

mATGL, mouse adipose triglyceride lipase.

**Table 2:** Primers for mATGL truncation and point mutations in eukaryotic expression vector pcDNA4/HisMaxC

Variant	Forward Primer 5′-3′	Reverse Primer 5′-3′	Vector
mATGL288	AGAGGAGGATTGATTGCAGCCTTATAGAAAAG	CCAGCCCTCTCCTCCACC	pcDNA4/HisMaxC
mATGL_FL_N209A	TCGCGTCACCGCTACCAGCATCC	AGCTCGTGGATGTTGGTG	pcDNA4/HisMaxC
mATGL_FL_I212A	CAACACCAGCGCTCAGTTCAACCTTCGCAATC	GTGACGCGAAGCTCGTGG	pcDNA4/HisMaxC
mATGL_FL_N215A	CATCCAGTTCGCTCTTCGCAATCTCTACCGC	CTGGTGTTGGTGACGCGA	pcDNA4/HisMaxC
mATGL_FL_ N209A/N215A	CCAGTTCGCTCTTCGCAATCTCTACCGCC	ATGCTGGTAGCGGTGACGCGAAGCTCGTG	pcDNA4/HisMaxC
mATGL_FL N209A/I212A/N215A	TCAGTTCGCTCTTCGCAATCTCTACCGCC	GCGCTGGTAGCGGTGACGCGAAGCTCGTG	pcDNA4/HisMaxC

mATGL, mouse adipose triglyceride lipase.

### Cloning of pST44-Trc3-mCGI58-His/Trc4-mATGL288-Str

The binary pST44-Trc3-mCGI58-His/Trc4-mATGL-Str construct was cloned by linearization of pST44-Trc3-smt3-TEV-GGG-mCGI58-His with BspEI and MluI as well as linearization of pST44-Trc4-smt3-TEV-GGG-mATGL288-Str with SacI and KpnI. The inserts required for ligation into translational cassette three and four of the linearized pST44 vectors were generated by complementary cutting of pST44-Trc3-smt3-TEV-GGG-mCGI58-His with SacI and KpnI and cutting of pST44-Trc4-smt3-TEV-GGG-mATGL288-Str with BspEI and MluI. Ligation reaction of the binary polycistronic pST44-Trc3-mCGI58-His/Trc4-mATGL-Str was performed with T4 DNA Ligase.

### Bacterial expression of recombinant mCGI-58, ATGL-proteins, mATGL/mCGI-58 complexes, and preparation of bacterial cell extracts

Expression of mATGL288 and mCGI-58 have been described before (5). Expression of mATGL288 protein and single amino acids exchange variants were performed in *Escherichia coli* ArcticExpress (DE3) cells (Agilent Technologies, Santa Clara, CA, USA) similar as previously described (5). Expression of the complexes mATGL288/mCGI-58, mATGL_N209A/mCGI-58, mATGL_I212A/mCGI-58, mATGL_N215A/mCGI-58, and mATGL_N209A/I212A/N215A/mCGI-58 complexes were also performed in *E. coli* ArcticExpress (DE3) cells. A day culture (LB media supplemented with 100 μg/ml ampicillin, 20 μg/ml gentamycin, and 2% glucose) was started and incubated for 8 h at 37°C. The day culture was transferred (1:50) to a preculture (with the same supplements as the day culture) for 8 h growth at 37°C. The main culture (LB media + 0.5% glucose) was inoculated (1:10) with preculture and grown at 30°C until it reached an *A*_600_ of 0.5. Cells were then induced with 0.1 mM of IPTG and harvested after 24 h of expression at 10°C. After harvesting the cells were frozen and stored at −20°C before further usage. All mATGL288 variants were treated equally.

Prior the activity assays, the cells for mATGL288 variants were resuspended in sucrose solution (250 mM sucrose, 1 mM EDTA, 1 mM DTT, 20 μg/ml leupeptine, 2 μg/ml antipain, 1 μg/ml pepstatin, pH 7) and disrupted by sonication on ice. After centrifugation at 15,000 *g* for 20 min at 4°C supernatants were collected. Total protein concentrations of the cell extracts were determined using the Bio-Rad Protein Assay Kit according to the manufacturer's instructions (Bio-Rad Laboratories, Hercules, CA, USA) using BSA as standard.

### Copurification of mATGL/mCGI-58 complexes

mATGL288 was coexpressed with mCGI-58 in ArcticExpress (DE3) cells. A 1 l cell pellet was thawed on ice and resuspended in 20 ml lysis buffer (100 mM K_2_HPO_4_/KH_2_PO_4_ pH 7.5, 100 mM KCl, 30 mM imidazole, 10% glycerol, 0.1% IGEPAL CA-630, 1 mM Tris(2-carboxyethyl)phosphine hydrochloride [TCEP], 10 mM ATP, 10 mM MgCl_2_) supplemented with 20 μl of 1,000× protease inhibitor stock solution (1.5 mM pepstatin A, 3.3 mM antipain, and 43 mM leupeptin). After cell lysis via sonication (10 min, cycle 5, 50% power) and centrifugation (40 min, 20,000 *g*, 4°C), the cell lysate was filtered using a 0.45 μm syringe filter. The chromatography steps were carried out using the ÄKTA Avant 25. As a first step, the lysate was loaded onto a 1 ml StrepTrap HP column (GE Healthcare Life Sciences, Buckinghamshire, UK) that was equilibrated with Lysis buffer.

The column was washed with 15 column volumes (CV) of lysis buffer, followed by 20 CV of nickel-wash buffer (100 mM K_2_HPO_4_/KH_2_PO_4_, pH 7.5, 500 mM KCl, 30 mM imidazole, 10% glycerol, 1 mM TCEP, 10 mM ATP, 10 mM MgCl_2_) and 20 CV of Strep-wash buffer (100 mM K_2_HPO_4_/KH_2_PO_4_, pH 7.5, 100 mM KCl, 10% glycerol, 1 mM TCEP). The proteins were eluted using 10 CV of a 100% Strep-elution buffer (100 mM K_2_HPO_4_/KH_2_PO_4_, pH 7.5, 100 mM KCl, 10% glycerol, 1 mM TCEP, and 10 mM desthiobiotin). Elution fractions were subjected to 12% SDS-PAGE to verify the presence of proteins. Samples with highest amounts of proteins were used for immunoblotting, using Strep-antibody against mATGL and His-antibody against mCGI-58. Of each variant a load (L), a wash (W), and an elution (E) sample were subjected to SDS-PAGE. “Load” stands for the cleared cell lysate which was loaded onto the column and “wash” is the last wash fraction taken before the elution (see also Supplemental Figures).

### Protein expression in Expi293F™ cells and preparation of cell extracts for TGH assays

His-mATGL and its point mutants were recombinantly expressed in Expi293F™ cells (Thermo Fisher Scientific, Waltham, USA). The cells were cultivated in Expi Expression Medium (Thermo Fisher Scientific, Waltham, USA) at standard conditions (37°C, 95% humidified atmosphere, 7% CO_2_). Cell density was determined in a CASY Cell Counter and Analyzer System (OMNI Life Science, Bremen, Germany). Cells in a total culture volume of 10 ml were transfected with 10 μg of plasmid DNA using the ExpiFectamineTM 293 Transfection Kit (Thermo Fisher Scientific) according to the manufacturer’s instructions. Two days after transfection, cells were harvested by centrifugation at 500 *g* and 4°C for 5 min. The cell pellet was washed twice with PBS (137 mM NaCl, 2.7 mM KCl, 10 mM Na_2_HPO_4_, 1.8 mM KH_2_PO_4_). Cells were disrupted immediately after harvesting by sonication (Sonopuls GM3100 equipped with a MS-72 tip; Bandelin, Berlin, Germany) in sucrose solution (250 mM sucrose, 1 mM EDTA, 1 mM DTT, 20 μg/ml leupeptin, 2 μg/ml antipain, and 1 μg/ml pepstatin) on ice. The homogenate was centrifuged at 1,000 *g* and 4°C for 20 min. The postnuclear fraction was collected and protein concentration was determined by the Bradford protein assay (Bio-Rad Laboratories, Hercules, USA) using (BSA) as standard.

### Purification of recombinant mouse mCGI-58

Purification of His_6_-smt3-mCGI-58 was performed via immobilized metal ion affinity chromatography as previously described (5). The concentration of the purified protein was determined via absorption at 280 nm.

### TG hydrolase assay

TG hydrolase activity assays was performed with some minor modifications as described elsewhere (5, 10, 38). To screen for the stimulation capacity of various mATGL288 variants, 25 μg protein of cell lysates containing ATGL were incubated together, and 1 μg purified mCGI-58 in a total volume of 25 μl sucrose solution (250 mM sucrose, 1 mM EDTA, 1 mM DTT, pH 7) with 25 μl ^3^H-triolein substrate. Empty uninduced ArcticExpress (DE3) cells were used as a negative control. TG substrate was prepared with 1.67 mM triolein, 25 μCi/ml [9,10-^3^H(N)]-triolein (PerkinElmer Life Sciences, Waltham, MA, US), and 188 μM L-α-phosphatidylcholine: L-α-phosphatidylinositol PC:PI (3:1) (Merck, Darmstadt, DE). 1.5 ml of 0.1 M potassium phosphate buffer, pH 7, was added and the mixture was subsequently sonicated. After sonication, FA free BSA (Merck, Darmstadt, DE) was added to a final concentration of 5%. As a blank measurement, 25 μl of sucrose solution were mixed with 25 μl of substrate solution (50 μl of reaction mixture). The protein-substrate mix was then incubated for 1 h at 37°C shaking. The reaction was terminated by adding 650 μl of methanol:chloroform:heptane (10:9:7) and 200 μl of 100 mM potassium carbonate buffer pH 10.5 (adjusted with boric acid). The radioactivity in 100 μl of the upper phase was determined by liquid scintillation counting. FA extraction was performed by mixing the samples vigorously for 5 s, followed by centrifugation at 2,400 *g* at room temperature for 10 min. An aliquot of 100 μl of the upper aqueous phase was transferred into scintillation vials containing 2 ml of scintillation cocktail (Roth). Radioactivity was determined by liquid scintillation counting using a MicroBeta Microplate Counter (PerkinElmer, Waltham, Massachusetts). Specific substrate activities were in the range of 1,000–2,000 cpm/nmol. Counts generated from blank measurements were subtracted and the rates of TG hydrolase activity, presented as nmol of released FA per hour and milligram of protein, were calculated according to Schweiger *et al.* (39). Calculations of EC_50_ values were done using approximation with sigmoidal function in Microsoft Excel.

### Immunoblotting

Bacterial lysates/purified proteins containing ATGL variants with point mutations and samples of mATGL288/mCGI-58 after copurification were loaded and run on a denaturing 12% SDS-PAGE gel and transferred to PVDF membrane (ROTH, pore size 0.45 μm) for 90 min at constant current of 220 mA. After the transfer, membranes were treated using standard protocols for Anti-His or Anti-Strep immunoblotting with minor modifications. For both anti-His and anti-Strep immunoblotting, standard TBS (50 mM Tris-HCl pH 7.5, 150 mM NaCl) and TBS tween buffer (TBST, 50 mM Tris-HCl pH 7.5, 150 mM NaCl, 0.2% v/v Tween 20) were used. For anti-His immunoblotting all membranes (mATGL288_WT/mCGI-58, mATGL288_N209A/mCGI-58, mATGL288_I212A/mCGI-58, mATGL288_I212S/mCGI-58, mATGL288_N215A/mCGI-58, mATGL288_N209A/I212A/N215A/mCGI-58) were blocked with 3% BSA in TBS buffer for 90 min at room temperature. Afterward they were washed 2 × 10 min in TBST and 1 × 10 min in TBS buffers accordingly. The PentaHis antibody (Qiagen, Düsseldorf, DE) against His-tagged proteins was diluted 1:5,000 in TBS buffer containing 3% BSA, and the membrane was incubated overnight at 4°C, followed by 2 × 10 min washing steps in TBST and 1 × 10 min in TBS buffers. The secondary antibody ECL anti-mouse IgG (GE Healthcare Life Sciences, Buckinghamshire, UK), diluted 1:5,000 in TBS buffer containing 5% milk powder was applied to the membrane for 90 min at room temperature. For anti-Strep immunoblotting all membranes (mATGL288_WT/mCGI-58, mATGL288_N209A/mCGI-58, mATGL288_I212A/mCGI-58, mATGL288_I212S/mCGI-58, mATGL288_N215A/mCGI-58, mATGL288_N209A/I212A/N215A/mCGI-58) were blocked with 3% BSA in TBST buffer containing 10% milk powder for 90 min at room temperature, followed by 2 × 10 min washing steps in TBST buffer. StrepMAB-Classic mouse antibody (IBA Lifesciences, Goettingen, DE) was diluted 1:2,000 in TBST buffer containing 5% milk powder, and the membrane was incubated overnight at 4°C. Next morning the membrane was washed 2 × 10 min in TBST buffer. The secondary antibody ECL anti-mouse IgG (GE Healthcare Life Sciences, Buckinghamshire, UK), diluted 1:5,000 in TBST containing 5% milk powder was applied to the membrane for 90 min at room temperature. Both anti-His and anti-Strep immunoblots were developed using Amersham ECL immunoblotting reagents (GE Healthcare Life Sciences, Buckinghamshire, UK, see also Supplemental Figures).

### Coimmunoprecipitation of proteins expressed in HEK293T cells

HEK293T cells were maintained in DMEM high glucose (Thermo Fisher Scientific, USA) at 37°C in a humidified atmosphere with 7% CO_2_. Cells were cotransfected with the indicated plasmid combinations using Metafectene (Biontex, Germany) according to the manufacturer’s instructions. Forty-eight hours later, cells were harvested in immunoprecipitation/lysis buffer (50 mM Tris-HCl pH 7.4, 150 mM NaCl, 1% NP-40, 1 mM EDTA, 20 μg/ml leupeptin, 2 μg/ml antipain, 1 μg/ml pepstatin) and incubated on a rotator at 4°C for 30 min. The samples were centrifuged at 14,000 *g* and 4°C for 20 min. Two milligrams of each lysate was incubated with 40 μl of Anti-FLAG M2 Affinity Gel (Sigma-Aldrich, USA) on a rotator at 4°C for 3 h. Beads were washed three times with 700 μl immunoprecipitation/lysis buffer. Bound proteins were eluted by boiling beads in 2× SDS sample buffer (0.1 M Tris-HCl pH 6.8, 5% 2-mercaptoethanol, 4% SDS, 20% glycerin, bromophenol blue) at 98°C for 10 min. For detection of protein expression, anti-Xpress (Thermo Fisher Scientific), anti-mouse-HRP Trueblot (Rockland), and anti-FLAG HRP (MiliporeSigma) antibodies were used (See also Supplemental Figures).

### Modeling of ATGL and the ATGL/CGI-58 complex using AlphaFold

ATGL and hetero multimers of ATGL with CGI-58 (ABHD5) were calculated using AlphaFold multimer. The predictions were based on either full-length protein from mouse (ATGL M1-C486; CGI-58 M1-351) or a truncated form of ATGL with a C-terminal truncation (ATGL M1-D264). Despite the differences, both predictions resulted in the identification of the same interaction interface. Furthermore, the same result was obtained when both combinations were predicted using AlphaFold monomer mode with an unstructured 50× glycine linker between the two different proteins. Predictions were either calculated with AlphaFold v2.1.2 full installation on a local workstation with following specifications: Nvidia GeForce RTX 3090 24 GB, AMD Ryzen Thread Ripper 3975WX using 48 cores and 192 GB RAM, or with AlphaFold v2.3.1 and local ColabFold v1.5.2 (40) installation using a workstation with Nvidia GeForce RTX 3090 24 GB, AMD Ryzen 95900X using 12 cores and 64 GB RAM.

## Results

### Evolutionary conservation provides an initial rationale for generating ATGL variants

Regulation of ATGL activity on a protein and activity level is very well studied with respect to the interaction of ATGL with CGI-58, G0S2, and HILPDA (10, 11, 12, 13, 14, 15, 16, 21, 22). Interestingly, the regulatory proteins are not conserved in all species, for example, the inhibitory protein G0S2 only exists in vertebrates while BLAST-searches of ATGL also reveal proteins with significant hits in nonvertebrates (15, 38). Therefore, we performed an unbiased computational screen to identify amino acids within the sequence of ATGL that mediate its interaction with regulatory proteins. We were primarily interested in the N-terminal half of ATGL since previous in vitro studies demonstrated that the truncated ATGL variants M1-L254 or M1-D288 can be activated by CGI-58 or inhibited by G0S2 and HILPDA (7, 9, 15, 19).

**Fig. 1:**
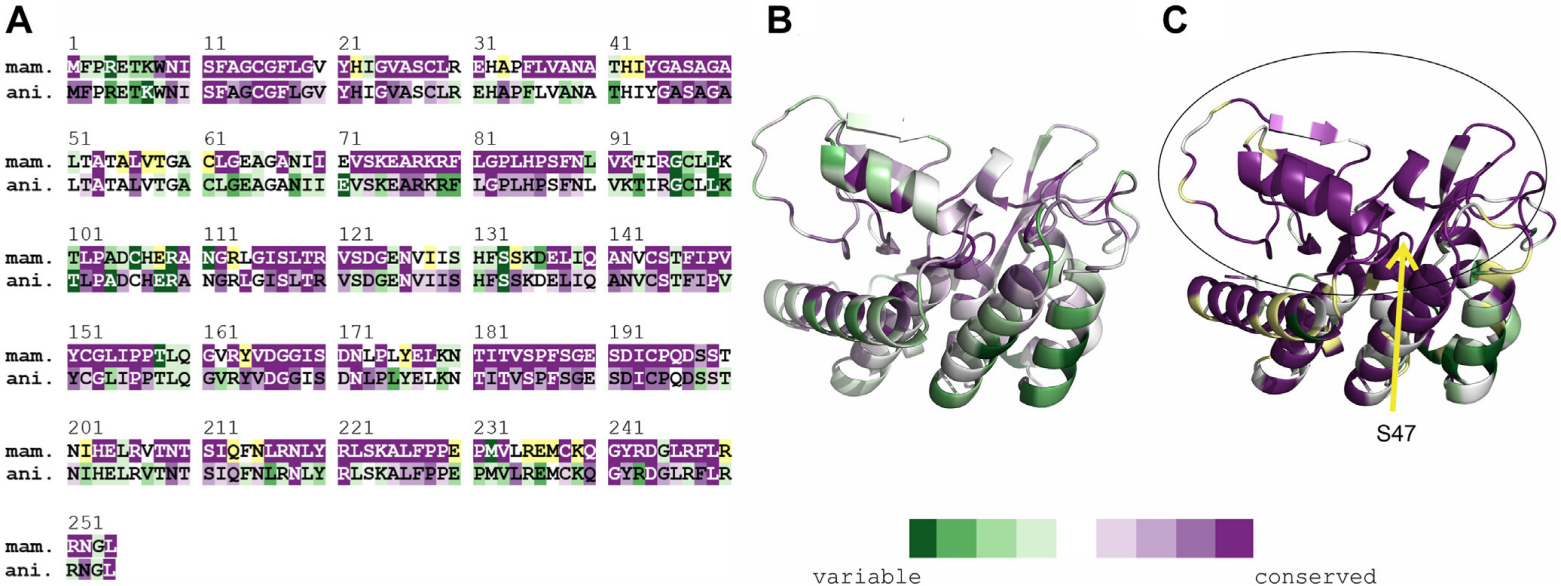
Evolutionary conservation of the minimal variant of mouse ATGL (mATGL254). The rate of conservation is color-coded dark green (variable) to violet (conserved). Residues with uncertainty in the conservation score are color-coded yellow. A: ConSurf analysis on a per-residue basis within the subgroup of mammals (mam.) compared to the conservation within animals (ani.) in general. B, C: ConSurf analysis plotted on the 3D model of mATGL254 represented in cartoon representation for (B) animals and (C) mammals. The position of the catalytic S47 is indicated with an arrow in (C). The prominent surface cluster of evolutionarily conserved sites in mammals is highlighted with an oval.

We compared the amino acid sequences of ATGL from different species, within the subgroup of “mammalia” and within a larger general group termed “animalia” including different chordates (fish, reptiles, birds, mammals) insects, nematodes, and mollusks. The analysis was carried out using the ConSurf software, a designated tool to identify functional regions in proteins by exploiting evolutionary data (Fig. 1A) (41). We hypothesized that catalytically and structurally essential regions are highly conserved, whereas regulatory regions might be *i*) less conserved when comparing mammalian ATGL with ATGL of nonmammalian species and *ii*) surface exposed to enable protein-protein interaction.

As shown in Fig. 1, the levels of conservation are quite different. Both groups, “mammalia” and “animalia” show large sequence variations in regions P3-K8, V57-C61, G96-T101, T158-Q160, and K179-N180. In line with our general hypothesis, the most conserved region even within “animals” is in the central core and catalytic region of the protein, whereas surface exposed parts of α-helices show higher sequence variability (Fig. 1B). Based on our hypothesis *i*) on sequence conservation, we identified regions F35-A40, V71-N89, V150-V165, F187-D197, H203-K229, and Y242 to match our criteria of conservation within “mammalia,” yet high diversity within “animalia.” To further narrow down residues of interest, we tested these residues for criteria *ii*) by plotting them on a 3D model of ATGL and analyzed the residues with respect to surface exposed residues. Accordingly, we decided to introduce the following single-amino acid exchanges: L81A, L84A, Y151A, Y164A, F187A, S188A, I193A, L205A, N209A, I212A, I212S, N215A, L216A, Y220A, R221A, L226A, F227A, Y242A. These residues are predominately surface-exposed and cluster on an almost continuous surface area of the protein (Fig. 1C). The amino acid exchanges were introduced in the C-terminally truncated variant of mATGL288 (5, 6, 15, 20, 38). We predominantly mutated aromatic and aliphatic amino acids residues since these side chains are frequently involved in biological interactions, whereas small alanine residues typically contribute very little to protein interactions. The exchange I212S alters the physicochemical properties more drastically by introducing a small, polar side chain.

### While some ATGL variants retain TG-hydrolytic activity and can be fully coactivated by CGI-58 others lose enzymatic activity and activatability

**Fig. 2:**
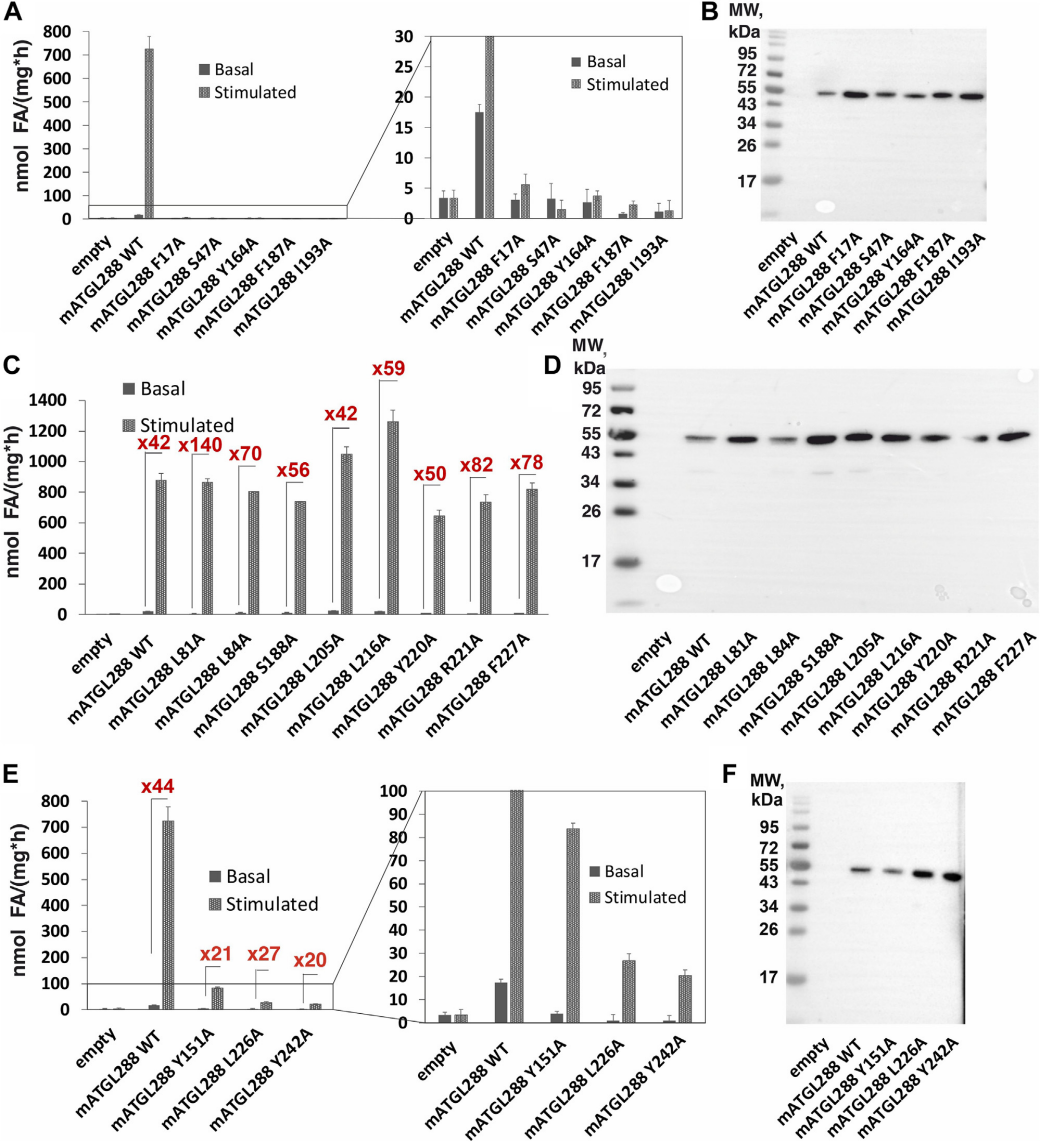
TG hydrolase activity assays and expression control of ATGL variants from bacterial lysates. mATGL288 WT enzymatic activity in basal and mCGI-58 coactivated condition was used as positive control; the multiplier for x-fold increase in activity is indicated for each variant. A: Inactive variants of mATGL288, namely F17A, S47A, Y164A, F187A, and I193A in comparison to WT mATGL288 under basal and mCGI-58 coactivated conditions based on enzymatic activity with zoom-in (right). B: Immunoblotting of the expression levels of inactive variants was used as expression control. C: Active variants of mATGL288 namely L81A, L84A, S188A, L205A, L216A, Y220A, R221A, and F227A under basal and mCGI-58 coactivated conditions. D: Immunoblotting analysis of the active variants. E: Partially active variants with residual enzymatic activity of mATGL288 namely Y151A, L226A, and Y242A. F: Immunoblotting of the expression levels of the partially active variants in comparison to WT mATGL288. Each TG hydrolase activity assay represents the assay of three technical replicates. At least two biological replicates were performed. ATGL, adipose triglyceride lipase; mATGL, mouse ATGL; TG, triacylglycerol.

Next, we expressed each ATGL288 variant in bacterial ArcticExpress (DE3) cells and tested the resulting lysate for TG hydrolase activities in absence or presence of recombinant CGI-58 (Fig. 2A, C, E and supplemental Figs. S1–S4). The expression levels of ATGL variants in transfected bacterial cells were assessed by immunoblot analyses (Fig. 2B, D, F). To clearly show the difference between basal activity and activatability, we give the X-fold multiplier by which activity is increased upon addition of CGI-58. The multiplier is also insensitive to slight changes in the expression level of ATGL, since basal activity and activity upon CGI-58 stimulation are equally affected. Amino acids F17 and the active site serine S47 are key amino acids for ATGL activity and the corresponding exchanges F17A and S47A served as negative controls (7, 31, 42).

We found that ATGL variants carrying the single amino acid substitutions F17A, S47A, Y164A, F187A, and I193A exhibit neither basal nor stimulated enzymatic activity (“inactive ATGL variants”) (Fig. 2A and supplemental Fig. S1). Our results also showed that ATGL variants carrying the single amino acid substitutions L81A, L84A, S188A, L205A, L216A, Y220A, R221A, and F227A had similar basal and stimulated TG hydrolase activities as WT ATGL (“active ATGL variants”) (Fig. 2C and supplemental Fig. S2). The variants Y151A, L226A, and Y242A exhibited significantly reduced but measurable basal activity compared to WT mATGL288 (“partially active ATGL variants”) (Fig. 2E and supplemental Fig. S3).

### ATGL variants N209A, I212A, I212S, and N215A have intact or slightly reduced basal activity, but lack effective coactivation by CGI-58

**Fig. 3:**
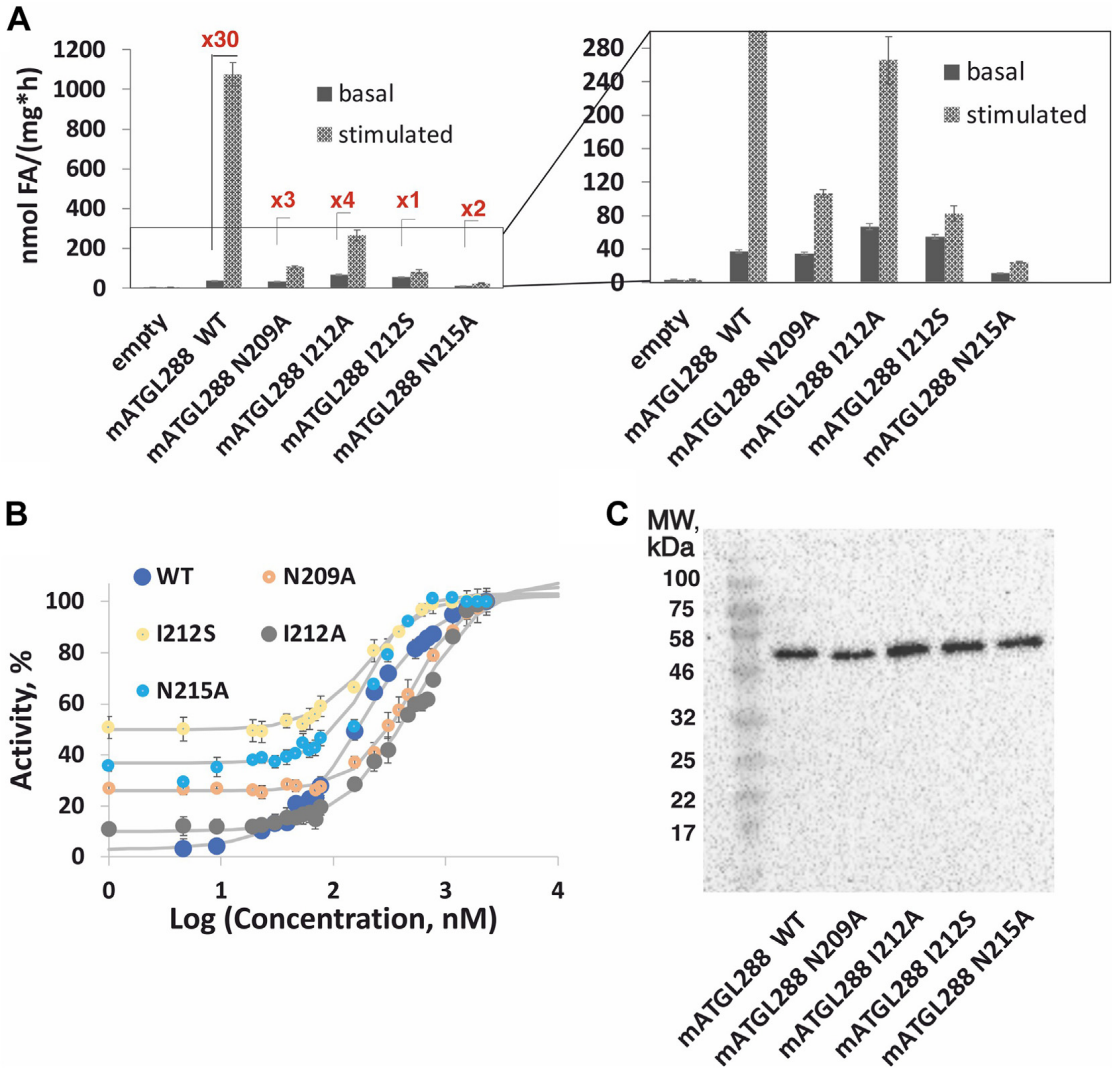
TG hydrolase activity assays of mATGL288 variants that cannot be effectively stimulated by mCGI-58. A: TG hydrolase assays of mATGL288 WT, N209A, I212A, I212S, and N215A under basal and mCGI-58 stimulated conditions from bacterial lysates. B: Dose-dependent coactivation of TG hydrolytic activity of mATGL288 variants by mCGI-58. Purified mCGI-58 protein was mixed with 25 μg mATGL lysate and tested in TG hydrolytic assays with different mCGI-58 concentrations. One hundred percent activity upon coactivation with 2,300 nM CGI-58 corresponds to 925 ± 30 nmol FA/(h∗mg) for mATGL288 WT, to 125 ± 4 nmol FA/(h∗mg) for mATGL288 N209A; to 352 ± 28 nmol FA/(h∗mg) for mATGL288 I212A, to 93 ± 10 nmol FA/(h∗mg) for mATGL288 I212S and to 60 ± 5 nmol FA/(h∗mg) for mATGL288 N215A. Data are presented as mean ± SD and are representative for three independent experiments. C: Expression controls of mATGL288 WT, N209A, I212A, I212S, and N215A were performed using immunoblot analysis. All ATGL variants were tested as bacterial lysates. ATGL, adipose triglyceride lipase; CGI, comparative gene identification; mATGL, mouse ATGL; PNPLA, patatin-like phospholipase; TG, triacylglycerol.

When we tested the N209A, I212A, I212S, and N215A variants, we observed intact or only slightly reduced basal activity, but reduced activability by mCGI-58 (approximately 1.5- to 4-fold) when compared to activation of WT mATGL288 by mCGI-58 (Fig. 3A and supplemental Fig. S4). To determine the EC_50_ of mCGI-58 to activate ATGL, we performed dose-dependent activity measurements of mATGL288 WT, N209A, I212A, I212S, and N215A with increasing concentrations of mCGI-58. The results revealed EC_50_ values in the range of 500 ± 50 nM for the N209A and I212A variants and 200 ± 20 nM for the I212S and N215A variants, in comparison to EC_50_ values of 178 ± 20 nM for WT mATGL288 (Fig. 3B). Immunoblots of the employed bacterial lysates indicated similar expression levels for WT ATGL and the ATGL variants (Fig. 3C).

### Plotting single amino acid exchanges on a 3D model of mATGL: N209, I212, and N215 are located on a surface region on one face of the protein

**Fig. 4:**
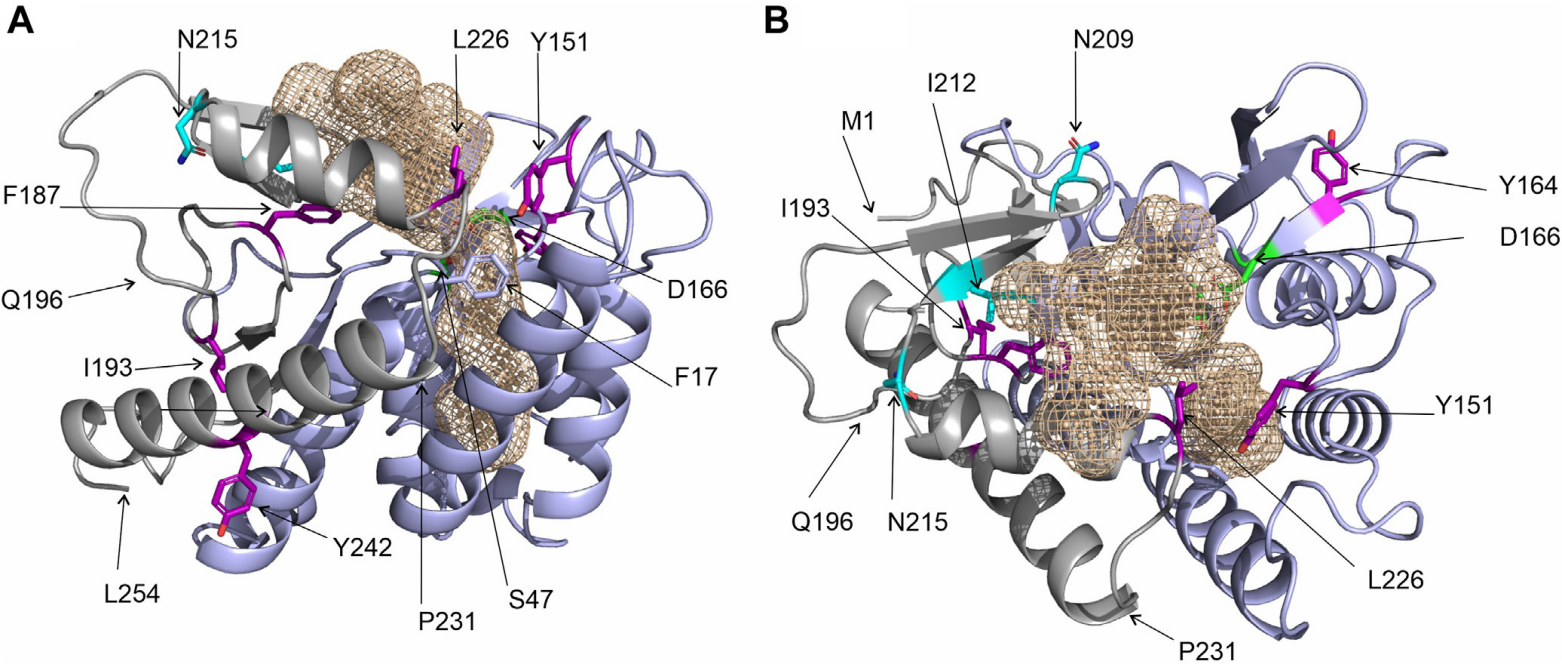
3D model of ATGL depicting important residues for activity and coactivation. The 3D structure of mouse ATGL (M1-L254) is in cartoon representation with the substrate binding cavity as a beige-colored mesh. The annotated PNPLA-domain (I10-K179), which is predominantly predicted with very high confidence, is colored light blue; residues M1-N9 and K179-L254 are in gray. The region (Q196-P231) is involved in shaping the entrance to the active site, yet is predicted with less confidence than the PNPLA-domain. Active site residues (S47, D166) are depicted as green sticks. A: Side-chains of Y151, Y164, I193, L226, Y242 are shown as purple sticks; N209, I212, and N215 as cyan sticks; F17 as light blue stick. The position of L254 is indicated. B: Cartoon representation of mATGL254 as in (A) after approx. 90° rotation to highlight the position of residues N209, I212, and N215 colored as cyan sticks. The positions of M1, Q196, and P231 are indicated with arrows. ATGL, adipose triglyceride lipase; mATGL, mouse ATGL; PNPLA, patatin-like phospholipase.

To locate the most crucial amino acid residues within the structure of ATGL, we generated a 3D model of mATGL254 using AlphaFold (Fig. 4). It is important to note that the model confidence varies significantly between different regions of the enzyme (5, 43). Almost the entire PNPLA domain and two short additional strands of the central β-sheet are modeled with very high confidence, namely residues W8–S73, I94–L173, and T181–P195 (confidence score “high”, >90). Similarly, P231–N252 form a long α-helix on the surface of mATGL254 that is predicted with very high confidence. In contrast, residues Q196–E230, which also show largest sequence variability (see Fig. 1A), are only predicted with “low” to “medium” confidence scores between 50 and 90. The residues N209, I212, and N215 are located within a short β-sheet of a beta-loop-beta region on the surface of ATGL; amino acid exchanges of those residues resulted in retained basal activity yet substantial loss of activability by CGI-58 (Figs. 3 and 4). Based on the 3D model of ATGL, this region forms a part of the substrate binding pocket that is distant from the catalytic site. We speculate that this region is not directly involved in the catalytic reaction at the scissile ester bond of the lipid, but plays a role in binding or release of the substrate or product (Fig. 4).

Substitution of the conserved aromatic residues F17, F187, and Y164 to alanine residues resulted in loss of ATGL-activity, which could not be rescued by addition of CGI-58. Amino acid exchanges Y151A, L226A, Y242A resulted in ATGL variants with reduced basal activity and reduced coactivation upon addition of CGI-58. In the 3D model of mATGL254 residues F17, Y151, F187, L226 are located in or at the entrance to the substrate binding pocket and might therefore not tolerate substitutions (Fig. 4). Y164 is positioned in a loop that helps in positioning of the catalytic residue D166 spatially close to S47. Thus, it might be essential for the formation of a principally functioning catalytic active site architecture within ATGL. Y242 is located on the surface exposed face of an α-helix. The complete loss of activity and coactivation upon introducing the change I193A might result from destabilization of ATGL due to loss of hydrophobic interactions (Figs. 2 and 4).

### Experiments with full-length ATGL confirm the relevance of N209A, I212A, and N215A for enzyme activation by CGI-58

**Fig. 5:**
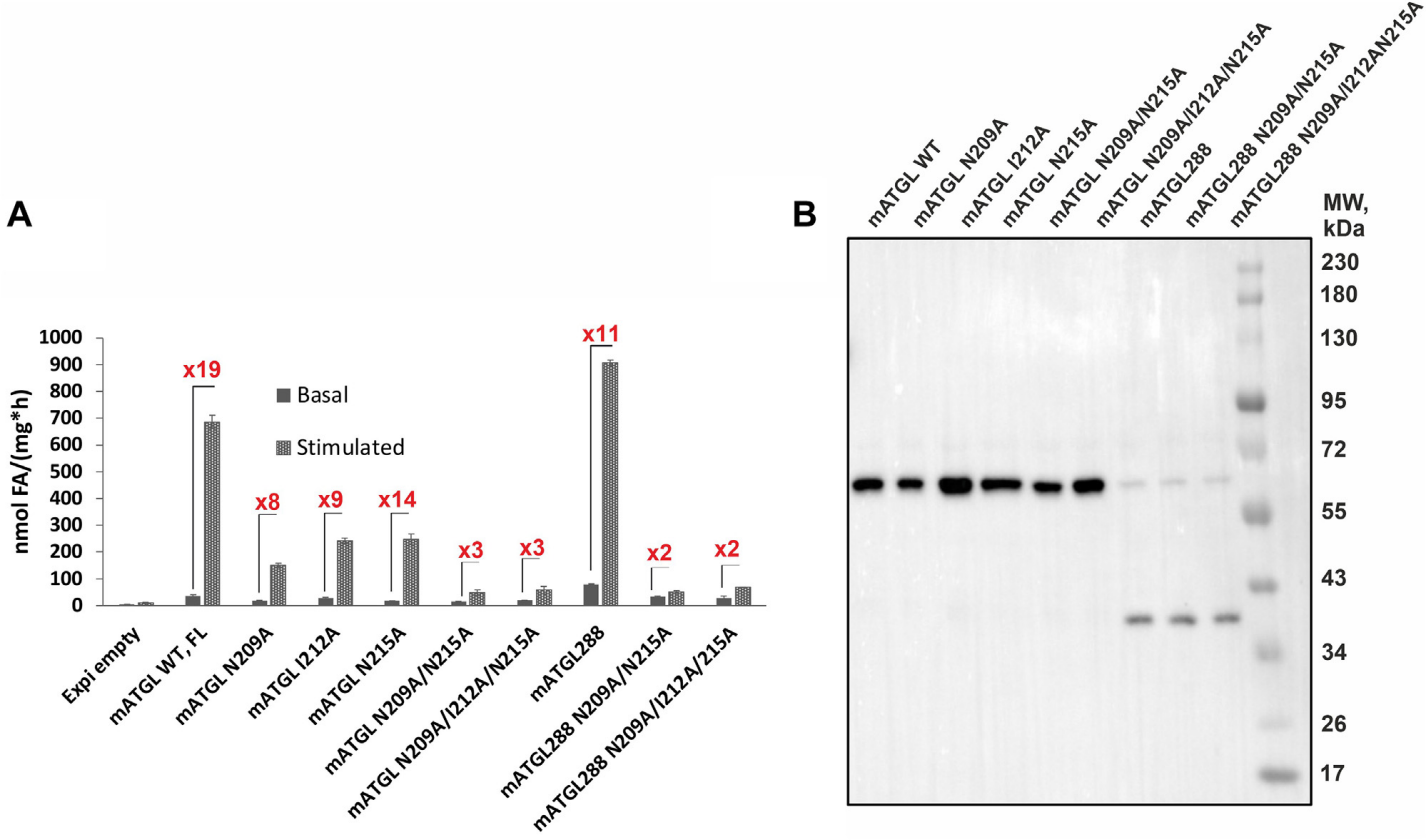
TG hydrolase activity assay of the ATGL variants expressed in mammalian Expi293F cells. A: Basal and mCGI-58 coactivated hydrolytic activity of mATGL in full-length background and in truncations after residue 288 (mATGL288) with single, double, and triple amino acid exchanges N209A, I212A, N215A, N209A/N215A, and N209A/I212A/N215A. B: Protein expression control of the mATGL variants was performed using immunoblot analysis. ATGL, adipose triglyceride lipase; CGI, comparative gene identification; mATGL, mouse ATGL; TG, triacylglycerol.

To investigate whether our results with bacterially expressed mATGL288 can be recapitulated with mATGL variants in lysates from a eukaryotic expression system, we transfected the suspension-adapted human embryonic kidney cell line Expi293F with WT and mutated mATGL variants and tested cell lysates for TG-hydrolytic activity (Fig. 5). Upon mCGI-58 addition, truncated WT mATGL288 was activated 11-fold, whereas the mATGL288 variants harboring double or triple amino acid exchanges, were only stimulated up to 2-fold (Fig. 5A). All mATGL288 variants showed similar expression efficacy in transfected Expi293F cells (Fig. 5B). In the eukaryotic system, we also tested full-length mATGL to confirm the results obtained with the truncated mATGL288 enzyme. Addition of CGI-58 increased TG hydrolase activity of lysates containing WT full-length ATGL approximately 20-fold. The single amino acid exchange variants N209A, N212A, and N215A were also activatable although to a lesser degree (8- and 14-fold). In contrast, double (N209A/N215A) or triple mutations (N209A/I212A/N215A) in full-length mATGL essentially lost the ability to be coactivated activated by mCGI-58 (Fig. 5A)) despite similar expression level (Fig. 5B).

### ATGL288 truncated variants that cannot be coactivated by CGI-58 preserve the ability to bind CGI-58

**Fig. 6:**
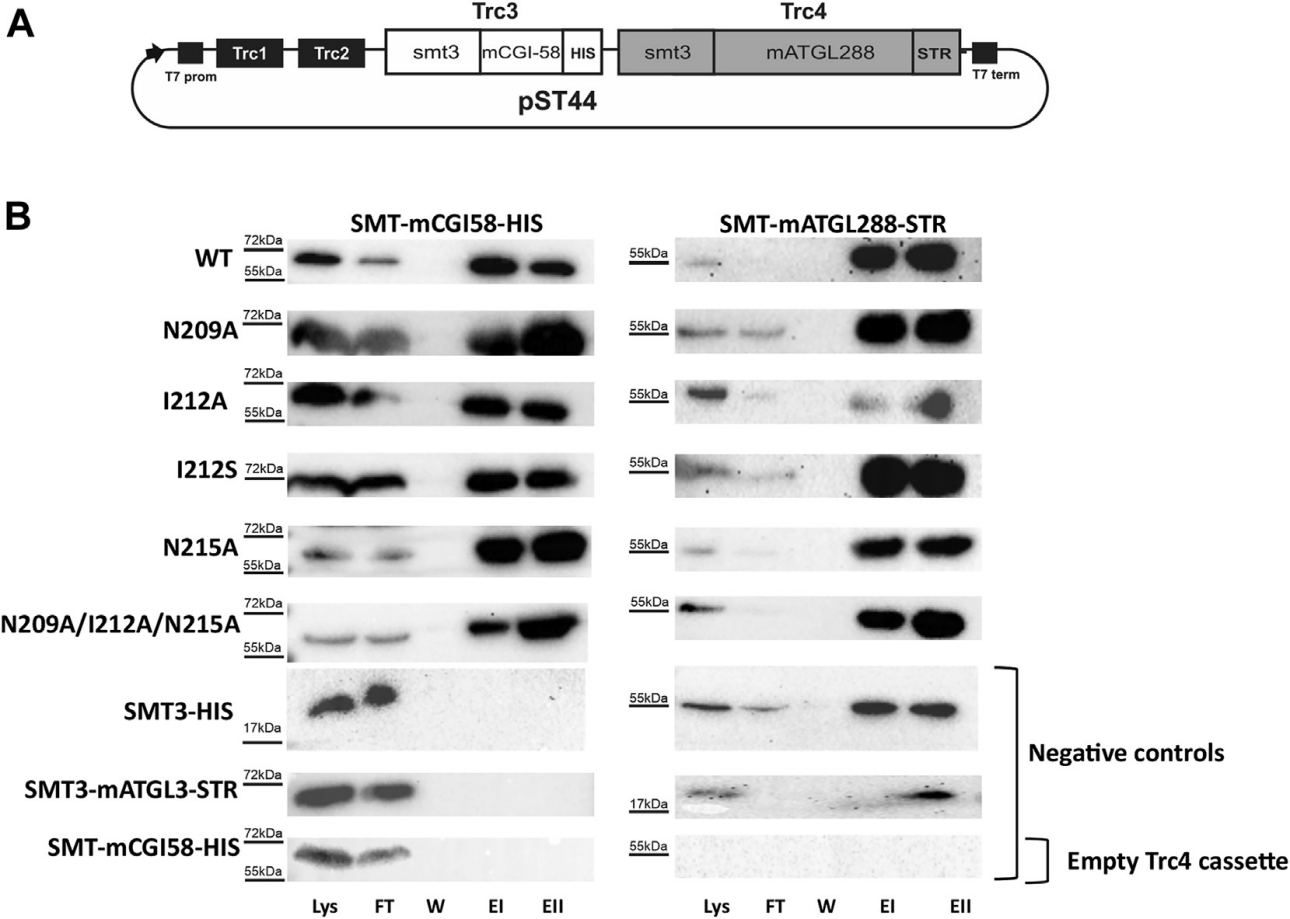
Copurification of mATGL288 variants with mCGI-58 using a Strep-II column. mATGL288 variants N209A, I212A, I212S, N215A, and N209A/I212A/N215A with ablated coactivation are still able to interact with mCGI-58. A: Schematic overview of the polycistronic pST44 vector system. Cassette Trc3 codes for SMT3-mCGI-58 variants with C-terminal His-tags, whereas cassette Trc4 codes for SMT3-mATGL288 with a C-terminal Strep-II tag. B: Immunoblot analysis of the affinity purification experiments from the coexpression of the pST44 vector coding different mATGL288 mutants and mCGI-58 and three different negative controls. mCGI-58 was detected with the anti-His antibody (left), whereas mATGL288 variant were detected with an anti-StrepII antibody (SMT3-mATGL288, right). The presence of proteins was monitored in fractions loaded onto the column (Lys), flow through (FT), last column wash (W), and two elution (EI and EII) fractions. CGI,-58 comparative gene identification-58; mATGL, mouse adipose triglyceride lipase ATGL.

Next, we addressed whether loss of activability of the mATGL288 variants N209A, I212A, I212S, N215A is a consequence of the enzyme’s inability to interact with mCGI-58. Therefore, we used the pST44 polycistronic expression system for coexpression of StrepII-tagged mATGL288 (smt3-mATGL288-StrepII) and variants thereof (N209A, I212A, I212S, N215A, and N209A/I212A/N215A) together with His-tagged mCGI-58 (smt3-mCGI-58-His6) (Fig. 6A). An analogous pST44 polycistronic expression system has been used before for testing ATGL/G0S2 interaction (38).

After cell lysis, we employed affinity purification utilizing a StrepII column, to isolate mATGL288 and potential mATGL288/mCGI-58 protein-protein complexes. Lysate and column fractions were analyzed for complexed and uncomplexed mATGL288 and CGI-58 by Western blotting analyses using anti-StrepII-antibody and anti-His-antibody for the detection of mATGL variants or mCGI-58, respectively (Fig. 6B): cell lysates were analyzed to demonstrate coexpression of the proteins; flow through to check for unbound protein; the last wash fraction (W) to verify that all unbound proteins had been removed after loading to the StrepII affinity column. Finally, two elution fractions (EI and EII) were tested for the presence of mATGL288/mCGI-58 complexes. Despite relatively low mATGL288-StrepII expression levels in the lysate, mATGL288-StrepII variants efficiently bound to the StrepII column and were easily detected upon elution (Fig. 3B, left). While mATGL288-StrepII was retained by the Strep column, uncomplexed His-tagged mCGI-58 expectedly passed through the StrepII-column (Fig. 3B, right). After extensive washing to remove all uncomplexed mCGI-58-His, StrepII-tagged mATGL288 and its complexes were eluted from the StrepII column with desthiobiotin. When we coexpressed the mATGL288 variants N209A, I212A, I212S, N215A, and N209A/I212A/N215A with mCGI-58, we observed a similar Lys/flow through/W/EI/EII pattern compared to mATGL288/mCGI-58 WT, suggesting that all mutant proteins bind to mATGL288 with similar efficiency. We utilized three negative controls to validate our findings. We coexpressed SMT3-mCGI-58-His in Trc3 with a severely truncated version of ATGL, that contained only three residues of ATGL (smt3-mATGL3-Str), essentially resulting in SMT3 with a Strep tag. As expected, this experiment indicated no binding with SMT3-mCGI-58-His (left) and confirmed the specific binding of SMT3-mATGL3-Str to the Strep-II resin (right). Second, coexpression of SMT3-His with SMT3-mATGL288-Str did not result in the elution of a complex. This demonstrates that there is no interaction of SMT3-His with mATGL288-Str and that there is no unspecific binding of SMT3-His to the Strep-II column. The third control had SMT3-mCGI58-His in the Trc3 cassette of the pST44 vector, but lacked any mATGL in the Trc4 cassette. This control demonstrated that mCGI-58 does not bind unspecifically to the Strep-II column (left) and confirms that the Strep-II antibody does not unspecifically detect mCGI-58 (right). These control experiments provide evidence that the observed elutions of mCGI-58/mATGL288 complexes were based on protein-specific interactions of mATGL288 with mCGI-58.

### Full-length ATGL variants closely mimic the retained capability to bind CGI-58 even after losing their coactivation property

**Fig. 7:**
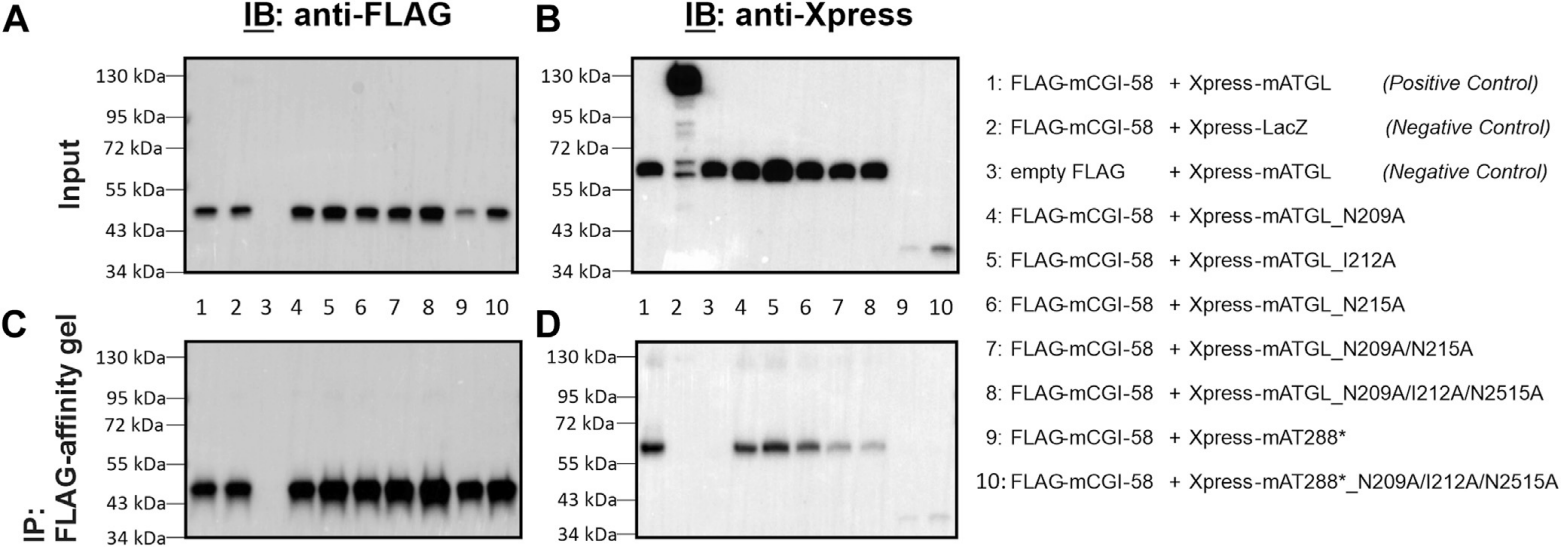
Coimmunoprecipitation analysis of FLAG-mCGI-58 and Xpress-mATGL proteins cotransfected in HEK293T mammalian cells. A: Immunoblot analysis confirming the expression of FLAG-tagged mCGI-58 protein and (B) Xpress-tagged mATGL protein. C: Immunoblot analysis confirming presence of coexpressed FLAG-mCGI-58 protein after coimmunoprecipitation with Xpress-mATGL variants using anti-FLAG HRP antibody. D: Xpress-mATGL variants after coimmunoprecipitation were detected with the anti-Xpress antibody. mATGL, mouse adipose triglyceride lipase.

To independently test the preserved binding ability of mATGL and mCGI-58, we performed coimmunoprecipitation studies in mammalian HEK293T cells. We employed coexpression of FLAG-tagged mCGI-58 and Xpress-tagged mATGL variants, including both, variants truncated after residue 288 and the full-length point-mutated variants N209A, I212A, and N215A, which were then coimmunoprecipitated to reveal mATGL288/mCGI-58 protein-protein complex formation. Protein expression control was carried out on HEK293T cell lysates to ascertain the presence of the proteins of interest using an anti-Xpress-antibody and anti-FLAG-antibody for the detection of mATGL variants or mCGI-58, respectively (Fig. 7A, B). The protein complexes that had formed were captured with anti-FLAG M2 magnetic beads and subsequently eluted through a boiling step. The eluted proteins were then subjected to immunoblot analysis for the detection of mATGL variants and mCGI-58 (Fig. 7C, D).

In Fig. 7A, B, we can observe high expression levels of full-length mATGL and mCGI-58. In contrast, the expression of mATGL288 and the mATGL288_N209A/I212A/N215A variant seems to be lower. Negative controls included FLAG-mCGI-58 coexpressed with Xpress-LacZ (as protein that does not bind to mCGI-58), as well as empty FLAG (no mCGI-58) coexpressed with Xpress-mATGL. After coimmunoprecipitation, it is evident that full-length mATGL variants, namely N209A, I212A, N215A, N209A/N215A, and N209A/I212A/N215A, as well as truncated mATGL288 WT and N209A/I212A/N215A variant, are bound to mCGI-58. This observation leads us to a crucial conclusion: even in the absence of coactivation ability, mCGI-58 maintains its binding affinity with mATGL.

### The in silico generated 3D model of the ATGL/CGI-58 complex supports the general interaction involving the region spanned by N209-N215 of ATGL and R299, G328, D334 of CGI-58

**Fig. 8:**
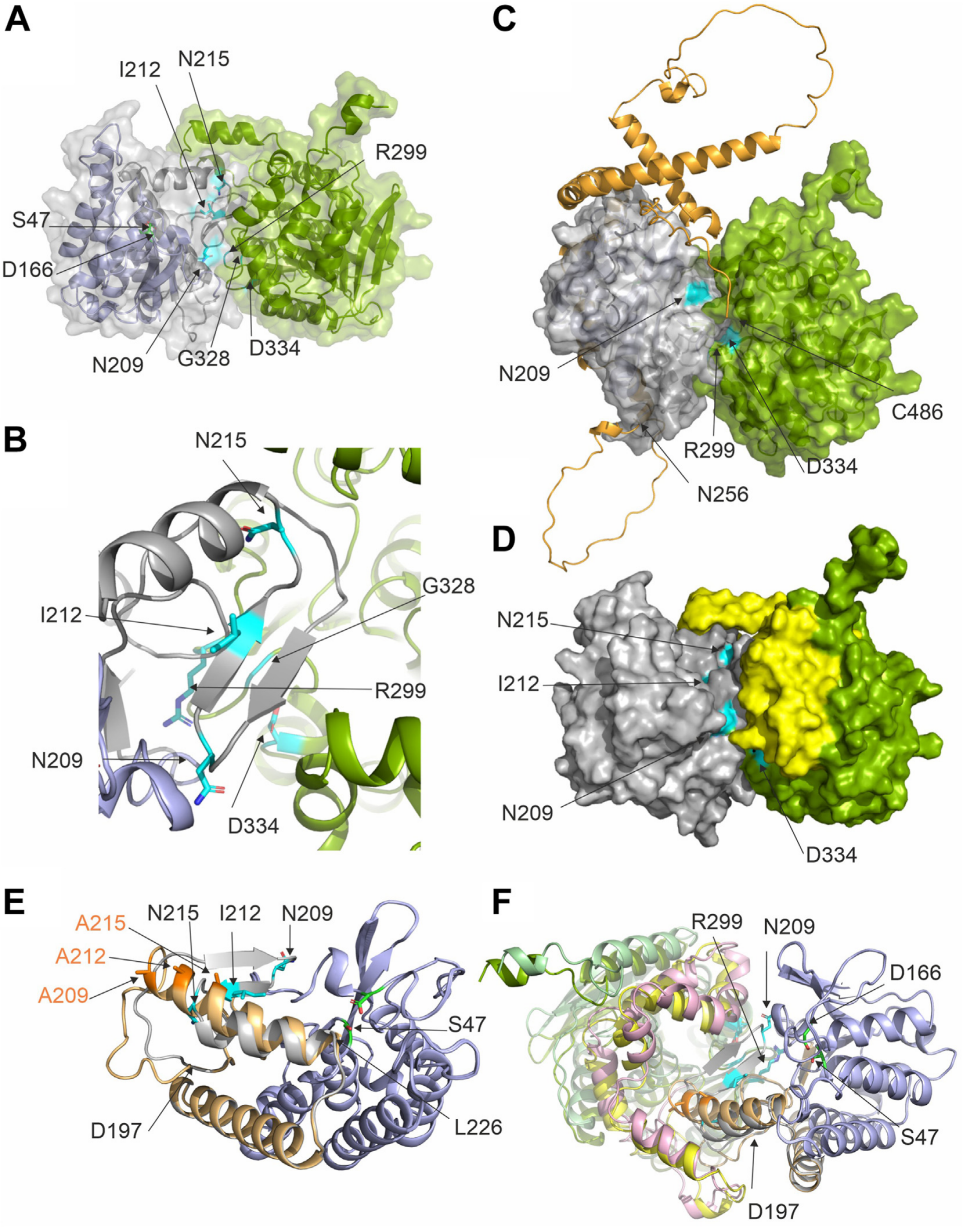
AlphaFold model of the ATGL/CGI-58 protein-protein complex. A–D: 3D models of mouse ATGL and mouse CGI-58 depicting important residues for activity and coactivation. The 3D structure of mATGL (M1-N259 in (A), M1-C486 in (C)) is in cartoon representation, the annotated PNPLA-domain (I10-L178), which is predominantly predicted with very high confidence, is colored light blue; residues M1-N9 and K179-L254 are in gray. Active site residues (S47, D166) are depicted as green sticks. mCGI-58 (G18-D351) is in pea-green cartoon representation. The transparent surface of both proteins is depicted in gray and pea-green, to highlight the extensive predicted protein-protein interaction surface. N209, I212, and N215 of ATGL as well as amino acids R299, G328, and D334 of CGI-58 are highlighted in cyan. B: Close-up view of the predicted interaction surface with N209, I212, and N215 of ATGL and amino acids R299, G328, and D334 of CGI-58 in cyan stick representation. C: 3D model of the ATGL/CGI-58 complex including the C-terminal half (N256-C486, colored in orange) of ATGL that is difficult to model with high confidence. D: Surface representation of the 3D model of the truncated ATGL/CGI-58 complex. The αβ-hydrolase core of CGI-58 is colored pea-green as in (A–C), yet the cap region of CGI-58 (P180-M279) is colored in yellow. E: Overlay of the 3D models of truncated WT ATGL with the N209A/I212A/N215A ATGL variant. Regions predominantly predicted with very high confidence are colored light blue; whereas regions K179-L254 with lower confidence in the prediction are in gray and sand for WT ATGL and N209A/I212A/N215A ATGL, respectively. ATGL is in similar orientation as in Figs. 1B, C, and 7F. F: Overlay of the complexes of WT ATGL/CGI-58 and that of the variant N209A/I212A/A215A ATGL with CGI-58. WT ATGL is colored light blue and gray; N209A/I212A/A215A ATGL is also in light blue, yet residues K179-L254 are in sand; the side chains A209, I212A, and A 215 are depicted as orange sticks. When CGI-58 is in complex with WT ATGL, it is colored as in (B), whereas CGI-58 in complex with N209A/I212A/N215A ATGL is colored in light green for the core and pink for the cap, respectively. ATGL, adipose triglyceride lipase; CGI-58, comparative gene identification-58; mATGL, mouse ATGL; PNPLA, patatin-like phospholipase.

Using AlphaFold, we generated an in silico model of the ATGL/CGI-58 complex (Fig. 8). For our analysis, we mapped the positions of N209, I212, and N215 of ATGL identified in this study and residues R299, G328, D334 of CGI-58 identified in previous work (16, 36). As seen in Fig. 8A–D, the interface between ATGL (truncated at residue 260 in this panel) and full-length mCGI-58 spans over large, yet continuous surface area of both proteins. I212 is central in a β-strand of a short two-stranded antiparallel β-sheet (E204-V207, T210-N213) at the far-end of the catalytic site in the predicted substrate binding pocket. N209 and N215 are located at the hinges of this β-strand. Substitution to of potential bidentate residues asparagine to alanine might change the flexibility of these hinges or be directly involved in changing protein-protein interaction (Fig. 3, Fig. 4 and 8). Importantly, data from experimental mutagenesis studies agreed very well with the calculated protein-protein interfaces. At the current stage of modelling, detailed analysis (e.g., with respect to intermolecular H-bonding, formation of salt bridges) or fine-tuned modeling of the protein-protein interaction interface requires additional data, preferably from an experimentally determined structure (Fig. 8B). The analysis of the C-terminus of ATGL is challenging, since large regions of the C-terminal part are modeled with very low, low, and at best medium confidences scores. Accordingly, the C-terminal part might adopt different secondary structures or different spatial positions in the physiological environment (e.g., the full LDs decorated with different LD-associated proteins, Fig. 8C). Next, we generated 3D models of truncated WT ATGL and the N209A/I212A/A215A ATGL variant. The triple variant exhibits large conformational rearrangements of region D197-L216, comprising a long loop and a short β-sheet in WT ATGL, by forming a loop and a long α-helix (Fig. 8E). This region coincides with the region of high conservational variability (Fig. 1) and low to medium confidence scores for structure prediction. It is interesting to note, that previous 3D homology modeling of the WT sequence of ATGL had an α-helix predicted for N209-L226, while the PNPLA domain (resides I10-K179) was essentially identical (44, 45). When we modeled the N209A/I212A/A215A ATGL/CGI-58 complex, the overall complex looked similar, however with some small changes in the interface. The model confidence for AlphaFold-Multimer models is described as an intrinsic score pTM and interface score ipTM (46). The truncated WT ATGL/CGI-58 model exhibited a combined pTM + ipTM of 0.78, while the triple variant truncated ATGL/CGI-58 model, which experienced conformational changes in the loop region at the interface, had a score of 0.68. These results suggest a somewhat weaker interaction between the two proteins. Nevertheless, the score remains relatively high, indicating a reasonably strong interaction. The model suggests an overall breathing motion and shifting of the coactivator CGI-58 to accommodate the longer α-helix and the lack of the short β-sheet of the ATGL variant (Fig. 8F). Together, these predictions indicate conformational flexibility beyond the PNPLA-domain and await further experimental insights of ATGL by itself and in complex with the coactivator CGI-58.

## Discussion

Intracellular lipolysis is a crucial metabolic process in energy homeostasis and diligent balance of its regulation leads to metabolic equilibrium. CGI-58 is a crucial regulator of ATGL activity, but the mechanism by which CGI-58 coactivates ATGL remains elusive. It is not known if binding of mCGI-58 affects the conformation of ATGL, facilitates substrate presentation, or increases the lipolytic activity of ATGL by removing reaction products from the active site, questions similar to the unknowns discussed for the G0S2-mediated inhibition mechanism of ATGL (38). We employed mutagenesis studies to identify specific amino acids in ATGL, which are required for its coactivation by CGI-58. The sites for amino acid exchanges were selected based on evolutionary conservation and surface exposure. For most experiments, we used a shortened version of mATGL, mATGL288, in combination with mouse full-length CGI-58 (mCGI-58) expressed in a bacterial expression system. Doing so, we could identify amino acids exchanges that resulted in complete loss of ATGL activity and exchanges leading to similar basal activity and activability by CGI-58 as observed for WT ATGL. Importantly, we identified the region N209-N215 of mATGL to play an essential role in mCGI-58–mediated coactivation of ATGL (Figs. 3 and 4). N209, I212, and N215 were recognized as residues with low evolutionary conservation (Fig. 1) and they turned out to be crucial residues for coactivation based on in vitro assays (Fig. 3). AlphaFold modeling revealed that these residues were also central for the mATGL288/mCGI-58 interaction in the interface of protein contacts (Fig. 8). The findings suggest that the N209-N215 region is not directly involved in executing the hydrolysis reaction but mediates CGI-58 coactivation of ATGL.

The comparison of ATGL variants from bacterial and mammalian expression system demonstrates that posttranslational modifications are not required for the coactivation of ATGL by mCGI-58 (Fig. 5). Furthermore, the comparison of full-length versus C-terminally truncated variants indicate that the full-length variants are slightly more tolerant toward single amino acid exchanges. This is reminiscent to our previous observations on full-length and truncated ATGL inhibition by variants of G0S2 (38). The 254 N-terminal amino acids of mATGL comprise the minimal domain that can be activated by mCGI-58 and inhibited by G0S2 (10). Supposedly, additional regions within the C-terminal half of ATGL together with other factors either affect the protein-protein interaction, or provoke conformational changes in the lipase, the coactivator or the LD-associated TG substrates. ATGL variants associated with neutral lipid storage disease are either enzymatically inactive proteins localizing to LDs or active TG hydrolases lacking LD localization (21, 47). Deletion of 220 amino acids from the C-terminus of human ATGL increases its interaction and activation by CGI-58 in vitro, in spite of defective LD localization in vivo in cultured cells (47). This finding indicates that the C-terminal region of ATGL is required for its targeting to LDs and plays an important (auto)regulatory role that requires further characterization.

When we modeled the structure of the ATGL/CGI-58 complex, we found that large surfaces of both proteins, ATGL and CGI-58, are predicted to be involved in protein-protein contacts. In our 3D model of ATGL residues N209, I212, and N215 are within in a beta-loop-beta region in an extension from the highly conserved PNPLA (I10-K179) domain on one face of the protein (Figs. 1 and 4). The predicted interface of ATGL is completely in line with the herein presented and previous experimental results. Literature reports on CGI-58 that identify regions and domains for CGI-58 also align well with the predicted interface presented here: the Trp-rich N-terminal region is not predicted to be involved in the interface, which is in agreement with its annotated role in serving as LD localization anchor (34). Both, the α/β-hydrolase core domain and the cap region (P180-M279, colored yellow in Fig. 7D, F) of CGI-58 are predicted to be involved in formation of a large protein-protein interaction surface. CGI-58 might not only aid in the localization of ATGL on the LD, yet also act as stabilizing scaffold for flexible parts of the beta-loop-beta region. The extent of the surface might also explain why the single and even triple amino acid exchanges did not totally abolish the interaction in copurification and coimmunoprecipitation assays (Figs. 6 and 7). The models of the ATGL WT and the variant ATGL_N209A I212A, I215A should be interpreted with special caution: The entire region N196-L222 (encompassing the beta-loop-beta region) has medium and low per-residue confidence (pLDDT) scores indicating structural and positional flexibility. There are different reports in the literature on AlphaFold’s capacity to predict the effects of single amino acid exchanges (48, 49, 50, 51). In the modeled ATGL/CGI-58 complex, the beta-loop-beta region harboring N209-N215 seamlessly merges into the pocket between the cap and the core of CGI-58. The model gives room for the hypothesis that diacylglycerols generated in the hydrolysis of TGs could exit ATGL via this beta-loop-beta region and could be ushered (even only temporarily) into the nonenzymatically active binding pocket between the cap and the core of CGI-58. If this beta-loop-beta region is lined with amino acids of different physico-chemical properties or even undergoes conformational rearrangement, the interaction and shuttling of the ligands/products is severely impaired. We find it promising that our 3D model of the ATGL/CGI-58 complex can effectively accommodate the predicted conformational changes for the mATGL_N209A/I212A/I215A variant while maintaining binding with CGI-58, the latter being experimentally confirmed in copurification coimmunoprecipitation experiments.

Previous studies had also demonstrated that CGI-58 interacts with fatty acid binding protein 4 (FABP4), which further promotes ATGL-mediated lipolysis and indicates the existence of an ATGL/CGI-58/FABP4 complex (52). In the model of the binary ATGL/CGI-58 complex, a direct transfer of hydrophobic ligands (substrates or products) from ATGL to CGI-58 appears possible (Fig. 8). AlphaFold-modeling of the binary CGI-58/FABP4 complex predicts that the helical portal region of FABP4 interacts with the cap region of CGI-58. This region of FABP4 has been shown experimentally to be involved in the FABP4/CGI-58 interaction using biochemical and biophysical methods and predicted interface residues of FABP4 (K32, V33, G35, M36, S56, F58, K59, D78, D77) experienced significant chemical shift perturbation upon titration with CGI-58 (52). Binding of long-chain FAs to FABP4 induces conformational changes in FABP4, especially in the so-called helix2 of FABP4 (F28-M36) and a loop harboring a crucially important phenylalanine (F58) (53). When we superimpose the predicted ATGL/CGI-58 complex with the predicted CGI-58/FABP4 complex, the entrance to the long-chain FA binding region of FABP4 is placed in proximity of the herein identified beta-loop-beta region containing N209-N215, which is important for coactivation of ATGL. Therefore, one can speculate that the release of long-chain FAs after the final step of the hydrolytic reaction is also aided via this beta-loop-beta portal of ATGL and the cap region of CGI-58.

In the absence of experimental 3D structures for ATGL and ATGL/CGI-58 complexes, our results represent a significant advance in our understanding of the protein-protein interactions formed by the ATGL/CGI-58 complex and provides novel insights into ATGL coactivation.

## Data availability

Data that support the findings of this study are presented in this article including the supplemental files. This study includes no data deposited in external repositories.

## Supplemental data

This article contains supplemental data.
