# Supplementary material for: Unmasking crucial residues in adipose triglyceride lipase for coactivation with comparative gene identification-58
Source: J Lipid Res. 2023 Dec 20;65(1):100491. doi: 10.1016/j.jlr.2023.100491 (PMC10828586; doi:10.1016/j.jlr.2023.100491)
Supplement: Supplemental Figures S1–S15 [file mmc1.docx]

**Supporting Information:**

***Unmasking Crucial Residues in Adipose Triglyceride Lipase (ATGL) for Co-Activation with Comparative Gene Identification-58 (CGI-58); Kulminskaya, Rodriguez Gamez et al., JLR***

**Figure S1.** TG hydrolase activity assays: mATGL288 WT enzymatic activity in basal (left) and mCGI-58 co-activated condition (right) for inactive variants of mATGL288, namely F17A, S47A, Y164A, F187A and I193A in comparison to WT mATGL288. The red dashed line in the right panel indicates the basal activity of mATGL288 WT. The value of co-activated activity of the mATGL288 wild type corresponds to 725 nmol FA/(mg*h).

**Figure S2.** TG hydrolase activity assays: mATGL288 WT enzymatic activity in basal (left) and mCGI-58 co-activated condition (right) for active variants of mATGL288, namely L81A, L84A, S188A, L205A, L216A, Y220A, R221A and F227A in comparison to WT mATGL288. The red dashed line in the right panel indicates the basal activity of mATGL288 WT.

**Figure S3.** TG hydrolase activity assays: mATGL288 WT enzymatic activity in basal (left) and mCGI-58 co-activated condition (right) for partially active variants of mATGL288, namely Y151A, L226A, and Y242A, in comparison to WT mATGL288. The red dashed line on the right panel indicates the basal activity of WT mATGL288. The value of stimulated activity of the WT mATGL288 corresponds to 725 nmol FA/(mg*h).

**Figure S4.** TG hydrolase activity assays: mATGL288 WT enzymatic activity in basal (left) and mCGI-58 co-activated condition (right) for variants with abolished stimulated activity of mATGL288, namely N209A, I212A, I212S and N215A, in comparison to WT mATGL288. The red dashed line in the right panel indicates the basal activity of WT mATGL288. The value of co-activated activity of the WT mATGL288 corresponds to 876 nmol FA/(mg*h).


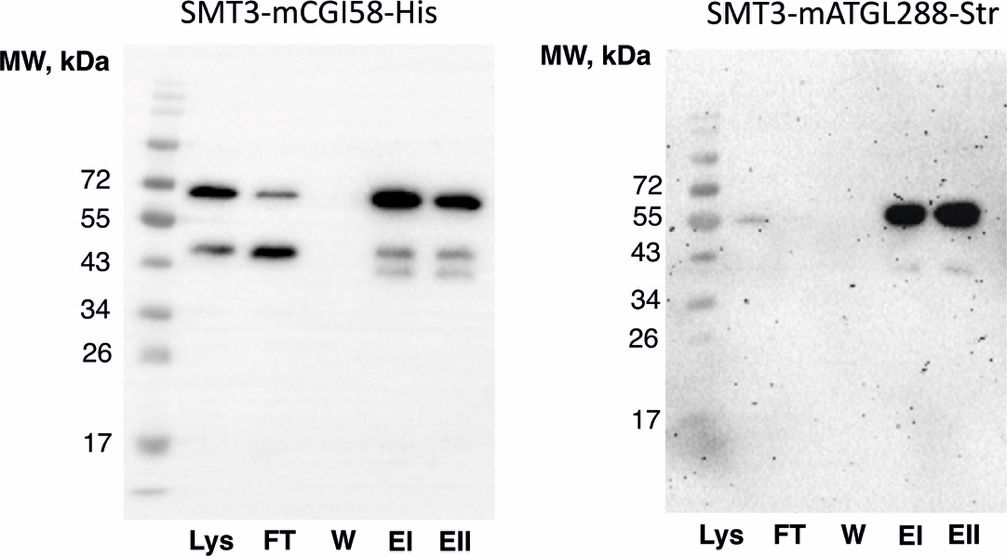


**Figure S5**: Immunoblot analysis of the affinity co-purification experiments from the co-expression of the proteins expressed in pST44 vector coding for mATGL288 WT and mCGI-58. mCGI-58 was detected with the anti-His antibody (left), whereas mATGL288 WT was detected with anti-StrepII antibody (SMT3-mATGL288, right). The presence of proteins was monitored in fractions loaded onto the column (Lys), flow through (FT), last column wash (W) and two elution (EI and EII) fractions. Please note that these images have the same source as used for Figure 6B in the main manuscript and are thus “re-used” images that are introduced in the supplementary file to show the full immunoblot.


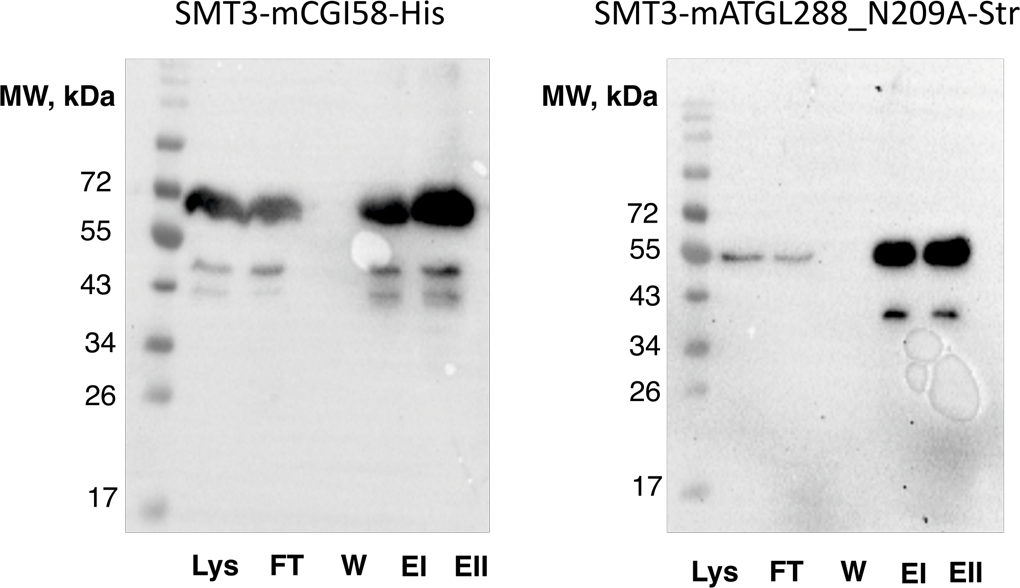


**Figure S6**: Immunoblot analysis of the affinity co-purification experiments from the co-expression of the proteins expressed in pST44 vector coding for mATGL288 N209A variant and mCGI-58. mCGI-58 was detected with the anti-His antibody (left), whereas mATGL288 N209A variant was detected with anti-StrepII antibody (SMT3-mATGL288, right). Presence of proteins was monitored in fractions loaded onto the column (Lys), flow through (FT), last column wash (W) and two elution (EI and EII) fractions. Please note that these images have the same source as used for Figure 6B in the main manuscript and are thus “re-used” images that are introduced in the supplementary file to show the full immunoblot.

**Figure S7**: Immunoblot analysis of the affinity co-purification experiments from the co-expression of the proteins expressed in pST44 vector coding for mATGL288 I212A variant and mCGI-58. mCGI-58 was detected with the anti-His antibody (left), whereas mATGL288 I212A variant was detected with anti-StrepII antibody (SMT3-mATGL288, right). Existence of proteins was monitored in fractions loaded onto the column (Lys), flow through (FT), last column wash (W) and two elution (EI and EII) fractions. Please note that these images have the same source as used for Figure 6B in the main manuscript and are thus “re-used” images that are introduced in the supplementary file to show the full immunoblot.


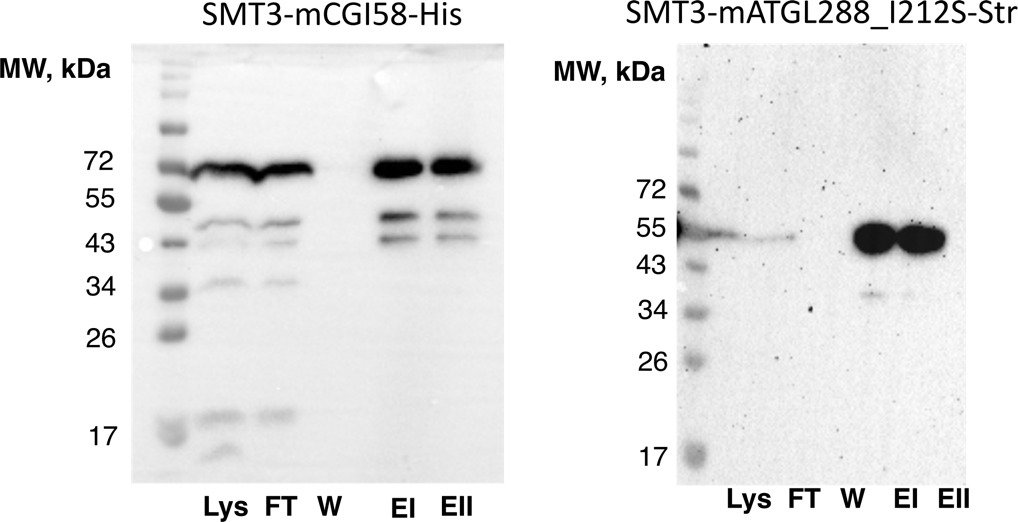


**Figure S8**: Immunoblot analysis of the affinity co-purification experiments from the co-expression of the proteins expressed in pST44 vector coding for mATGL288 I212S variant and mCGI-58. mCGI-58 was detected with the anti-His antibody (left), whereas mATGL288 I212S variant was detected with anti-StrepII antibody (SMT3-mATGL288, right). Existence of proteins was monitored in fractions loaded onto the column (Lys), flow through (FT), last column wash (W) and two elution (EI and EII) fractions. Please note that these images have the same source as used for Figure 6B in the main manuscript and are thus “re-used” images that are introduced in the supplementary file to show the full immunoblot.


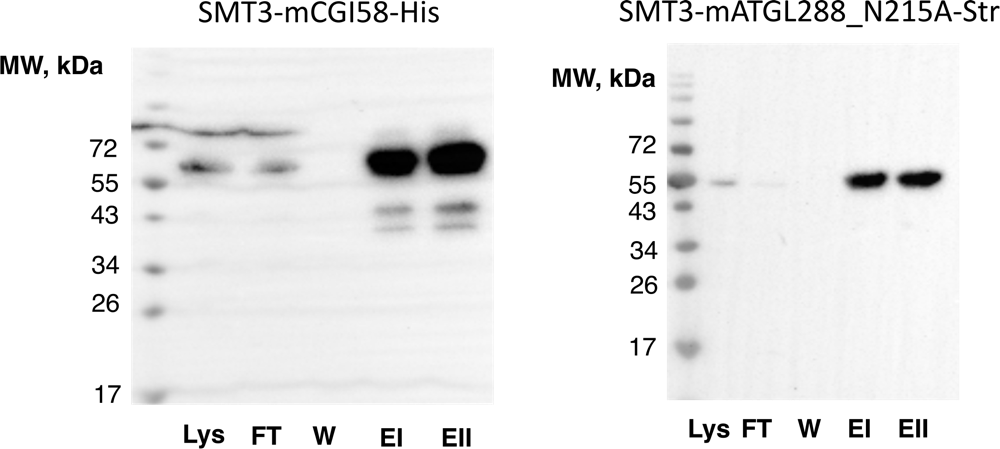


**Figure S9**: Immunoblot analysis of the affinity co-purification experiments from the co-expression of the proteins expressed in pST44 vector coding for mATGL288 N215A variant and mCGI-58. mCGI-58 was detected with the anti-His antibody (left), whereas mATGL288 N215A variant was detected with anti-StrepII antibody (SMT3-mATGL288, right). Existence of proteins was monitored in fractions loaded onto the column (Lys), flow through (FT), last column wash (W) and two elution (EI and EII) fractions. Please note that these images have the same source as used for Figure 6B in the main manuscript and are thus “re-used” images that are introduced in the supplementary file to show the full immunoblot.


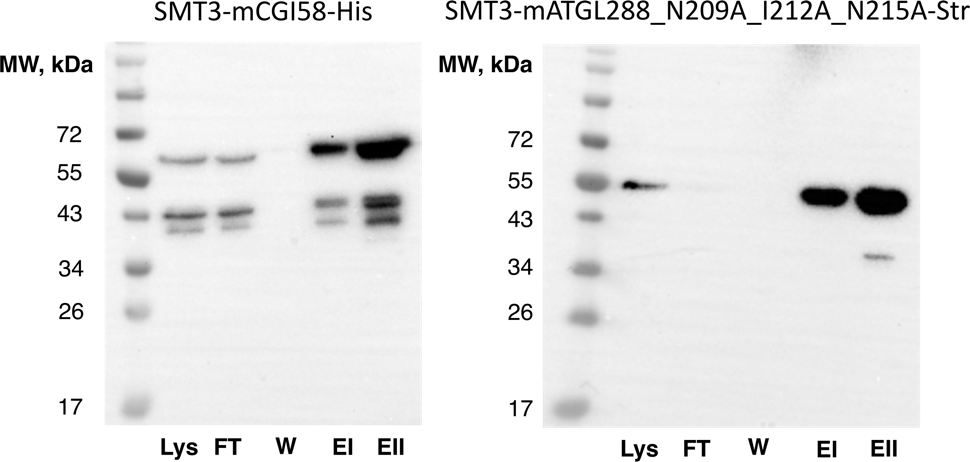


**Figure S10**: Immunoblot analysis of the affinity co-purification experiments from the co-expression of the proteins expressed in pST44 vector coding for mATGL288 N209A_I212A_N215A variant and mCGI-58. mCGI-58 was detected with the anti-His antibody (left), whereas mATGL288 N215A variant was detected with anti-StrepII antibody (SMT3-mATGL288, right). Existence of proteins was monitored in fractions loaded onto the column (Lys), flow through (FT), last column wash (W) and two elution (EI and EII) fractions. Please note that these images have the same source as used for Figure 6B in the main manuscript and are thus “re-used” images that are introduced in the supplementary file to show the full immunoblot.


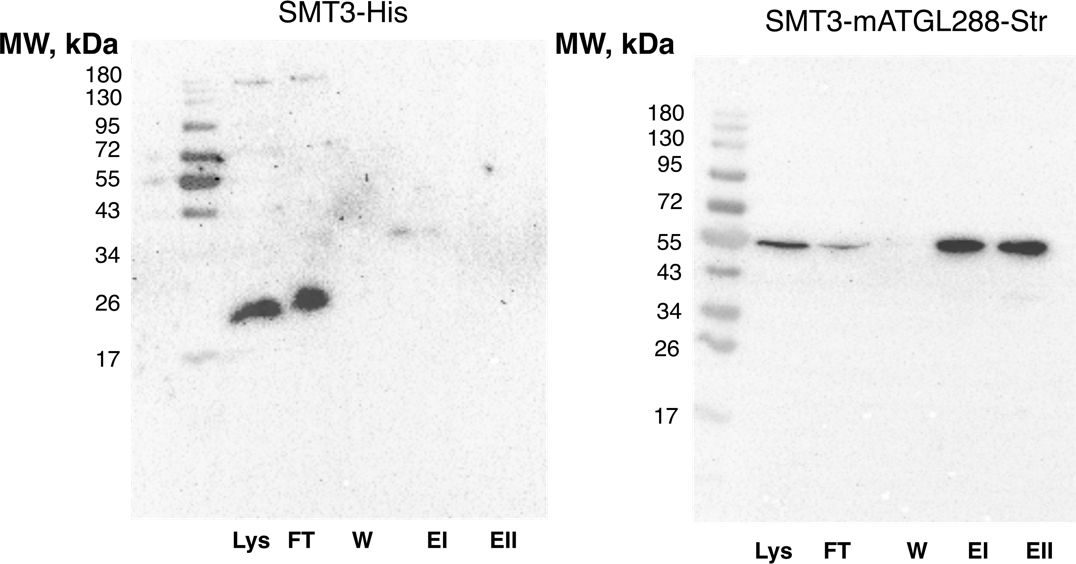
­­

**Figure S11**: Negative control: Immunoblot analysis of the affinity co-purification experiments from the co-expression of the proteins expressed in pST44 vector coding for SMT3 variant and mATGL288. SMT3 was detected with the anti-His antibody (left), whereas mATGL288 variant was detected with anti-StrepII antibody (SMT3-mATGL288, right). Presence of proteins was monitored in fractions loaded onto the column (Lys), flow through (FT), last column wash (W) and two elution (EI and EII) fractions. Please note that these images have the same source as used for Figure 6B in the main manuscript and are thus “re-used” images that are introduced in the supplementary file to show the full immunoblot.


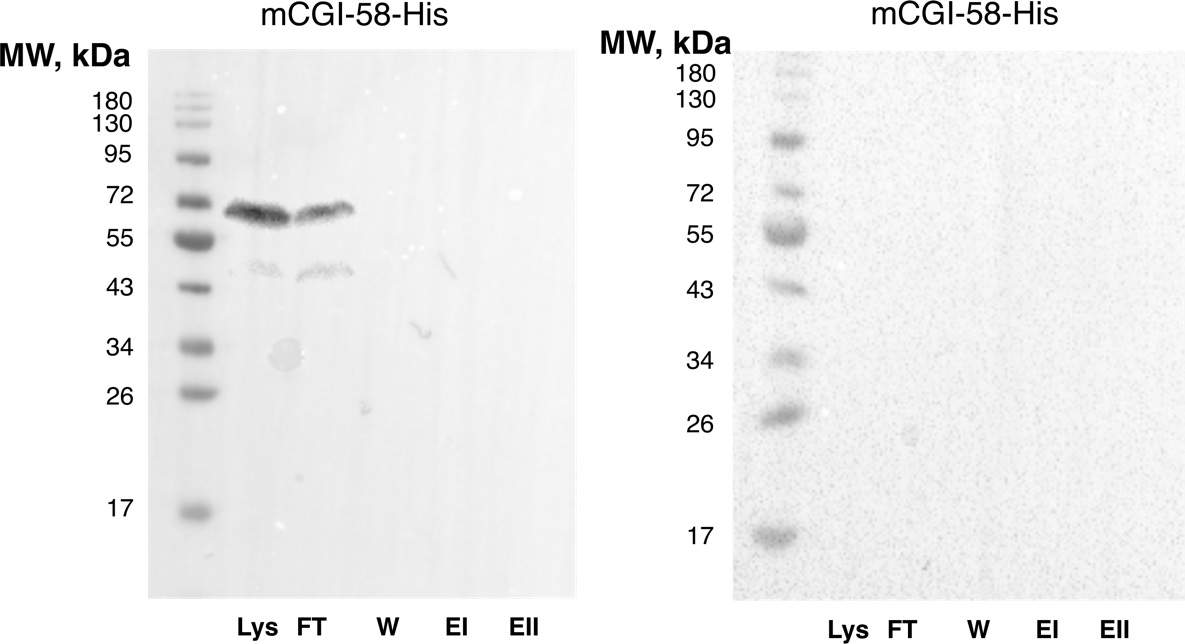


**Figure S12**: Negative control: Immunoblot analysis of the affinity purification experiment from the His-tagged mCGI-58 protein expressed from Trc3 of the pST44 vector after purification using StrepII column and detection using anti-His antibody (left) anti-Strep-II antibody (right). Please note that Trc4 is empty. Presence of proteins was monitored in fractions loaded onto the column (Lys), flow through (FT), last column wash (W) and two elution (EI and EII) fractions. Please note that these images have the same source as used for Figure 6B in the main manuscript and are thus “re-used” images that are introduced in the supplementary file to show the full immunoblot.


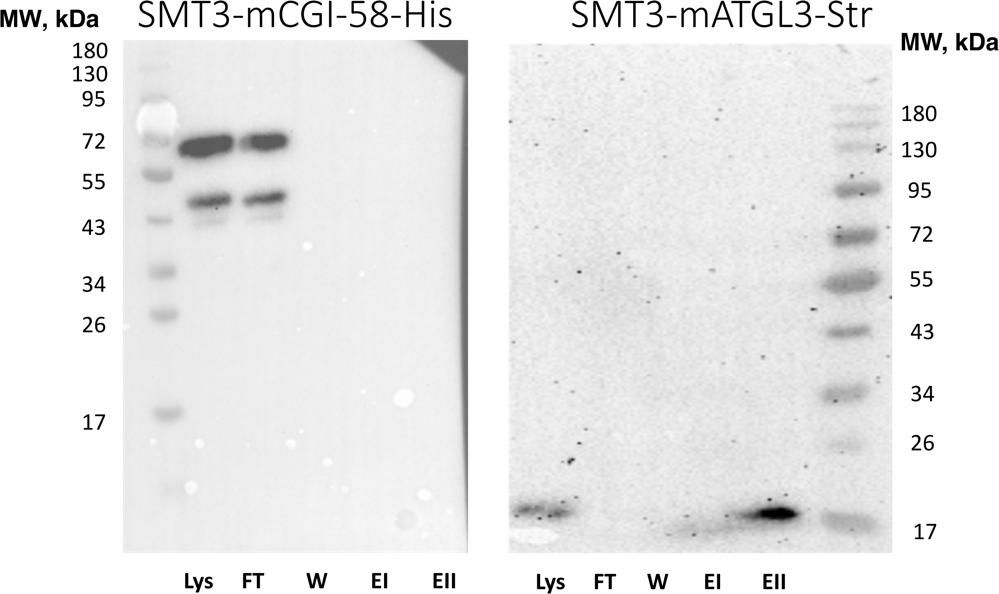


**Figure S13**: Negative control: Immunoblot analysis of the affinity co-purification experiments from the co-expression of the proteins expressed in pST44 vector coding for mATGL3 variant and mCGI-58. mCGI-58 was detected with the anti-His antibody (left), whereas mATGL3 variant was detected with anti-StrepII antibody (SMT3-mATGL3, right). Protein of interest was monitored in fractions loaded onto the column (Lys), flow through (FT), last column wash (W) and two elution (EI and EII) fractions. Please note that these images have the same source as used for Figure 6B in the main manuscript and are thus “re-used” images that are introduced in the supplementary file to show the full immunoblot.


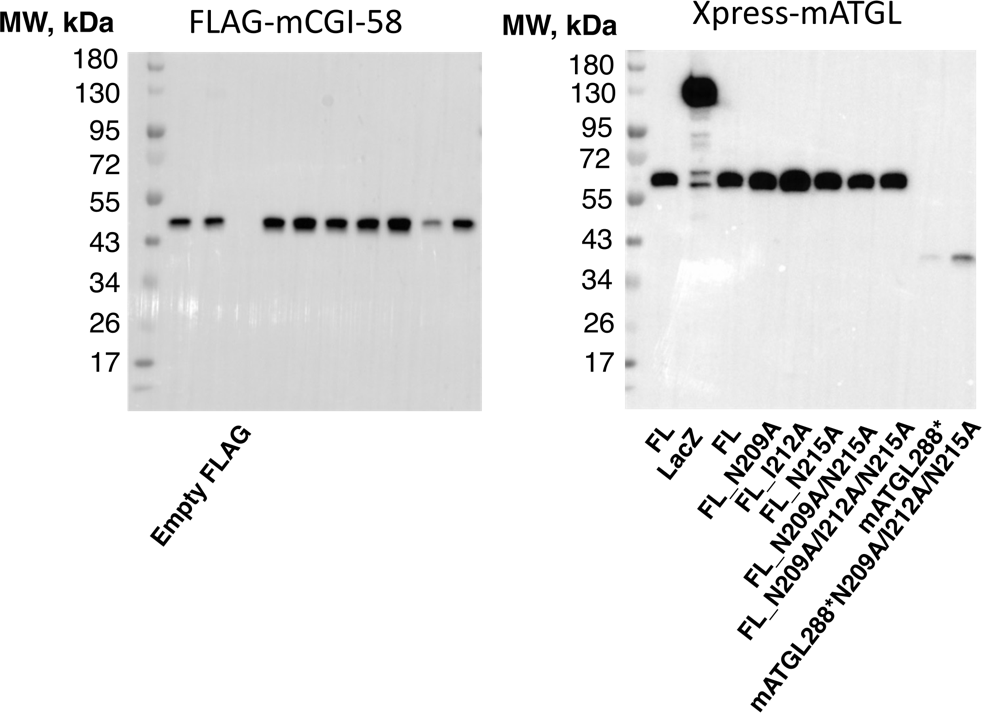


**Figure S14**: Immunoblot analysis showing the expression control of FLAG-mCGI-58 and Xpress-mATGL proteins expressed in HEK293T cells. mCGI-58 was detected with the anti-FLAG antibody (left), whereas mATGL variants were detected with anti-Xpress antibody (right). Please note that these images have the same source as Figure 7A and 7B in the main manuscript and are thus “re-used” images that are introduced in the supplementary file to show the full immunoblot.


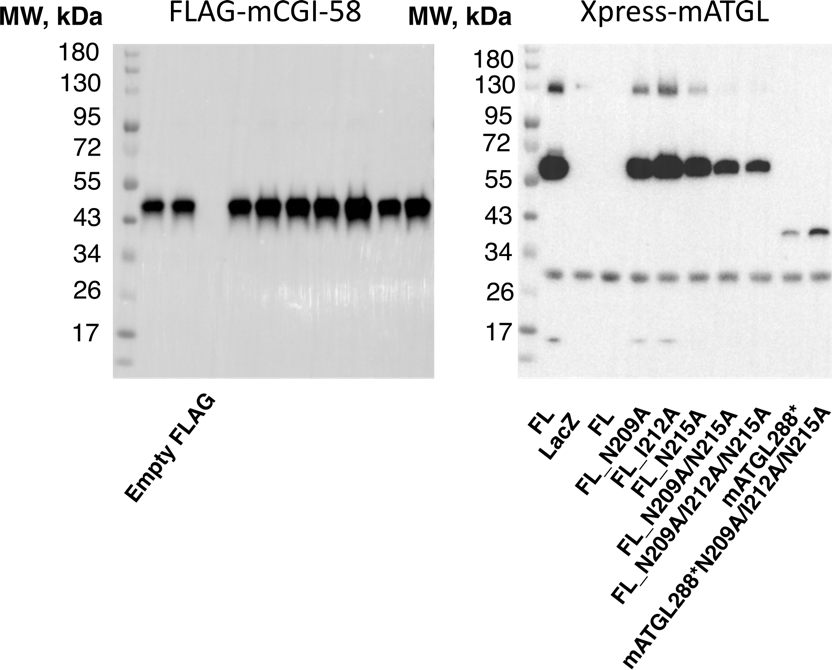


**Figure S15**: Immunoblot analysis showing co-immuno-precipitation of FLAG-tagged mCGI-58 and Xpress-tagged mATGL proteins expressed in HEK293T cells. mCGI-58 was detected with the anti-FLAG antibody (left), whereas mATGL variants were detected with anti-Xpress antibody, 40 s accumulation mode (right). Please note that these images have the same source as Figure 7C and 7D in the main manuscript and are thus “re-used” images that are introduced in the supplementary file to show the full immunoblot.
